# The Effects of Aspartame on Glucose, Insulin, and Appetite-Regulating Hormone Responses in Humans: Systematic Review and Meta-Analyses

**DOI:** 10.1016/j.advnut.2025.100449

**Published:** 2025-05-15

**Authors:** Lucy R Boxall, Fatemeh Eskandari, Julie Wallis, Aleksandra D Bielat, Katherine M Appleton

**Affiliations:** Department of Psychology, Faculty of Science and Technology, Bournemouth University, Poole, United Kingdom

**Keywords:** aspartame, E951, low-calorie sweeteners, nonnutritive sweeteners, glucose, insulin, appetite-regulating hormones, energy intake, appetite, adverse events

## Abstract

Aspartame (L-α-aspartyl-L-phenylalanine methyl ester) has been implicated in increased risk of several chronic health conditions, yet underlying mechanisms remain unclear. The objective of this work was to systematically identify and summarize all controlled intervention studies investigating the effects of aspartame consumption on glucose, insulin, and appetite-related hormone responses. Five academic databases, 4 trial registries, and additional resources were searched until June 2024. Search hits were screened, in duplicate, for intervention studies of aspartame compared with comparator, which assessed glucose, insulin, and/or any other appetite-regulating hormone. Results were tabulated, and meta-analyses run where ≥10 studies with similar methodology were found. Risk of bias (RoB) was assessed using RoB-2. Certainty of the evidence was assessed using Grading of Recommendations Assessment, Development, and Evaluation. One hundred one articles were identified, detailing 100 experiments: 79 acute (≤1 d), 8 medium term (2–30 d), and 13 long term (>30 d). Experiments involved healthy adults, individuals with aspartame sensitivity, and individuals with compromised glucose metabolism, varied widely in aspartame provision and comparator/s, and although almost all assessed glucose and/or insulin responses, few experiments investigated other appetite-regulating hormones. Meta-analyses (acute cross-over studies) revealed few effects of aspartame on blood glucose/insulin compared with vehicle or low-calorie sweeteners (LCS), and lower blood glucose/insulin concentrations compared with sugars, other carbohydrates, or other nutritive elements. Over the medium term and longterm, few effects of aspartame were found, and high heterogeneity between studies remained. Similar effects were found in other populations, and other outcomes, with few adverse events. RoB assessments suggested “some concerns” for the majority of studies. The certainty of the evidence for all outcomes in all populations was judged to be “very low.” Our findings suggest little to no effects of aspartame consumption on glucose metabolism over the short term or the long term. Further studies over the long term, assessing a range of appetite-regulating hormones and comparing aspartame with other LCS, would be of value.

This study was registered in PROSPERO as CRD42024540781 on April 29, 2024.

**Statement of significance**

Although the health impacts of aspartame consumption remain controversial, this work identified 100 experiments investigating the effects of aspartame consumption on glucose, insulin, and other appetite-regulating hormone responses. Little to no effects of aspartame were found over the short term or long term, with no contraindications for health.

## Introduction

A high consumption of free sugars is associated with increased energy intake, raising risk for overweight, obesity, and various chronic conditions, including type 2 diabetes, cardiovascular disease, and metabolic syndrome [1,2]. Given these associations, the WHO currently recommends limiting free sugar intakes to 10% of total energy intake [2], with added health benefits if consumption is reduced below 5% [2]. One strategy for reducing free sugar intakes is food and drink reformulation [[2], [3], [4]]. Whether for financial [4] or consumer-orientated reasons [5], reformulation involves reducing the sugar content of food and drink products [[3], [4], [5], [6]]; an action achieved by many food manufacturers through increased use of low-calorie sweeteners (LCS) [4,6].

LCS provide the pleasure of sweet taste in the absence of or for a much reduced use of sugars [7,8], allowing manufacturers to retain the desirable sweet taste of foods and drinks traditionally high in free sugars [9], although reducing their sugar content. LCS are commonly recognized as safe [10,11], widely used within the food industry [10,11], and recent reviews suggest benefits for reducing energy intake and body weight (BW), when compared with the consumption of sugar [[12], [13], [14]]. Over the long term and for chronic health conditions, however, benefits of LCS consumption are less clear. LCS consumption has been associated with increased risk of obesity, several metabolic conditions [13,[15], [16], [17], [18], [19]], and some other adverse events [[19], [20], [21]], although the evidence available is limited and largely stems from cohort studies, which can suffer from bias [13,19].

Effects of LCS on energy intake and BW are considered to result from the reduced energy content of LCS when compared with sugar [[12], [13], [14]]; a feature of all LCS [7,8]. However, effects on other health conditions are thought to result, at least in part, from disruptions to sugar and sweet food metabolism, including effects on blood glucose, as achieved via the actions of a number of appetite-regulating hormones [18,19]. Although all LCS provide a sweet taste for reduced energy, for effects on metabolism, consideration of the differing physiological actions of differing LCS may be required. Differing LCS have different chemical structures resulting in different physiological activities, both within and beyond the oral cavity [19,22,23].

One of the most commonly used LCS is aspartame (L-α-aspartyl-L-phenylalanine methyl ester) [7,24]; an LCS known to be entirely metabolized on consumption by the human digestive system [22,23]. Aspartame is a chemical LCS, ∼200 times sweeter than sucrose, recognized as safe as a food additive (E951), for use in a variety of foods and beverages, such as drinks, desserts, sweets, dairy products, chewing gum, low-calorie and weight control products, and as a table-top sweetener [[25], [26], [27]]. Following consumption, aspartame is broken down into methanol, aspartic acid, and phenylalanine [22,23], each of which is then metabolized as from other dietary sources [22,23]. Considering this complete breakdown to metabolites that are also found elsewhere in the diet, metabolic effects as a result of aspartame, as an LCS, may seem unlikely. Some studies, however, suggest differing effects, for a range of health outcomes, from aspartame consumption compared with those of other LCS [20,28], and controversy over aspartame use continues [7,24,29,30].

This work aimed to investigate the effects of aspartame on glucose responses, insulin responses, and responses in any other appetite-regulating hormone. Reviews on LCS as a group suggest few systematic differences in the effects of different LCS on appetite and/or hormone responses [18,23,[31], [32], [33]], but few studies for each LCS are typically included. More recent reviews have focused on individual LCS, including aspartame [34,35], but few studies have contributed to these. Given the limited number and nature of the studies in these reviews, conclusive findings are difficult to draw.

This work aimed to systematically identify and summarize all controlled intervention studies investigating the effects of aspartame consumption on glucose, insulin, and appetite-related hormone responses. Simultaneous effects on appetite, energy intake, and adverse events were also considered, where these were measured.

## Methods

This systematic review with meta-analyses followed the PRISMA statement [36]. Objectives, eligibility criteria, and methods for analysis were specified and registered in advance on PROSPERO: registration ID: CRD42024540781, registration date: April 29, 2024 [37]. We adhered to our registered protocol in all respects, with the following exceptions: searches for unpublished works were not undertaken; for assessments of risk of bias (RoB), we used the Cochrane Collaboration RoB-2 tool [38], rather than the original Cochrane Collaboration RoB criteria [39]; in addition to assessments of RoB, assessments of the certainty of the evidence were undertaken using the Grading of Recommendations Assessment, Development, and Evaluation (GRADE) approach [40,41]; and meta-analyses were undertaken only where ≥10 comparable studies were available, as detailed below in the section Data Synthesis.

### Searches

Systematic searches to identify all articles investigating the effects of aspartame on glucose, insulin, and appetite-regulating hormone responses were carried out. Five academic databases were searched: PubMed, Medline, CINAHL, Web of Science, and the Cochrane Library; 4 trial registries: clinicaltrials.gov, the WHO International Clinical Trials Registry Platform, the Australian and New Zealand Clinical Trials Registry, and the International Standard Randomised Controlled Trial Number registry; and all publicly available European Food Safety Authority (EFSA) and US Food and Drink Administration (FDA) submissions for regulatory purposes. For academic databases, 1 search string was used, composed of terms relating to aspartame combined with (AND) terms relating to glucose, insulin, and appetite-regulating hormones. Terms were searched for in “title” and “abstract” fields, overall years of records. The detailed search strategy is presented in the Supplemental Materials. Trial registries and EFSA and FDA databases were searched using only the search terms related to aspartame and were again searched overall years of records. Searches were set to include conference proceedings, conference abstracts, book chapters, and any other type of publication and were not limited by language, but were limited to “humans,” where this restriction was permitted.

Database searches were supplemented by searching reference lists of published review articles and all included articles, for any study that may have been missed. Our searches aimed to identify as many articles, and as much data, as possible, of relevance to our research questions.

### Study inclusion

Inclusion and exclusion criteria for studies to be included in the review were developed based on population, intervention, comparator, outcomes criteria, with additional information on study design. For each category:

Population: We included studies involving human participants of any age, gender, or ethnicity, who were healthy, with overweight or obesity, or with impaired glucose metabolism (i.e., prediabetes, diabetes type 1 or 2, impaired glucose tolerance). Studies of individuals with medical or clinical conditions, other than those related to glucose metabolism, were not included.

Intervention/exposure: We included studies involving all types of aspartame consumption — alone, in water, in conjunction with other nutrients or foods, in combination with other LCS, in tablet form or capsule form. We included studies using any dose, including if dose was unspecified, any pattern of consumption, e.g., single or repeated exposure, and whether aspartame was included in the original study as the intervention or comparator. Studies were included only if the use of aspartame was confirmed by authors if this was unclear from the publication, e.g., some experiments described the use of an LCS-sweetened drink without detailing the specific LCS in the original publication. We excluded studies where consumption of aspartame could not be confirmed. Studies were included regardless of duration of the exposure and regardless of repetition.

Comparator: Comparator arms must have used the same vehicle without inclusion of aspartame, without the inclusion of aspartame with an alternative LCS, or without the inclusion of aspartame with a caloric sweetener or other nutritive element (e.g., sucrose, glucose, or any sugar alcohol, including the glucose provided for an oral glucose tolerance test), with assessments reported over the same time frame or at the same time points, as for the aspartame arm. In acute closely controlled settings, studies were included only if other aspects of the intervention that may influence physiological responses were controlled, including nutritive components, beverage flavorings, and outcome assessment patterns; we excluded studies where the aspartame condition and comparator condition differed by more than the aspartame. For long term studies conducted in real-world settings, small differences between intervention and comparator, e.g., beverage flavorings, were permitted, to more accurately reflect the real-world scenario. Long term studies were excluded if differences between the aspartame condition and comparator condition were known to influence digestive responses, e.g., where an aspartame-sweetened beverage was compared with a milk beverage or a multivitamin beverage.

Outcomes: Primary outcomes of interest were glucose responses, insulin responses, and other appetite-regulating hormone responses, and outcomes must have been assessed using objective, validated measures. Studies were excluded from the review if they did not measure glucose responses or any appetite-regulating hormone or if they did not assess these using objective validated measures. Secondary outcomes were energy intake, appetite (e.g., hunger, satiety, fullness), and adverse events. Secondary outcomes were only considered in the studies that were identified as investigating our primary outcomes; we did not search for these outcomes. These outcomes must also have been assessed using objective, validated measures.

Study design: Any controlled intervention study design (within-groups cross-over or between-subjects parallel-groups) was considered suitable, provided empirical data were included. Studies were included regardless of setting, location, or date of study. Animal studies, in vitro studies, and observational studies were excluded.

### Study selection

Searches were undertaken by 1 reviewer (L.R.B.). Search results were downloaded into Endnote, and duplicates were removed. Titles and abstracts were assessed independently by 2 researchers (L.R.B. and A.D.B.), and all articles of possible relevance to the review were taken forward for full-text screening. Screening of trial registries was undertaken by 2 reviewers (L.R.B. and F.E.), and searching for references from published reviews was undertaken by 1 reviewer (F.E.). Screening of all full-text articles was subsequently undertaken by 2 independent reviewers (L.R.B. and J.W.). Discrepancies were resolved by discussion or following consultation with a third reviewer (K.M.A.). Where the use of aspartame was unclear, clarity of this was sought from authors by e-mail (F.E.). All articles for which authors provided details that aligned with our inclusion and exclusion criteria were subsequently included.

### Data extraction

Two reviewers (L.R.B., F.E., J.W., or A.D.B.) independently extracted data from all included articles using a bespoke data extraction spreadsheet. Data were extracted on methodological aspects of each study and RoB. Discordances were discussed and resolved between reviewers. Following the extraction of all methodological and RoB details, numerical data were also extracted by 2 independent reviewers (L.R.B., F.E., or J.W.) for all studies to be included in the meta-analyses. Only group-level data were extracted; individual data were not sought. Data were extracted directly from publications and subsequently converted to means and standard deviations (SDs) as required. For the extraction of numerical data located in graphs, the online tool Plotdigitizer (www.plotdigitizer.com) [42] was used. Numerical data points were compared between 2 researchers, with all extracted data within 5% automatically accepted and an average taken. Where extracted data were not within 5%, these were manually compared and agreed by consultation. For studies not to be included in meta-analyses, results were extracted narratively (F.E. and K.M.A.) for inclusion in the review alongside the numerical results.

### Risk of Bias

Two reviewers (L.R.B., F.E., J.W., or A.D.B.) independently extracted data on RoB for each included study using the Cochrane Collaboration RoB-2 tool, developed by Sterne et al. [38]. The domains assessed were: *1*) RoB arising from the randomization process; *2*) RoB due to deviations from the intended interventions (effect of assignment to intervention and effect of adhering to intervention); *3*) RoB due to missing outcome data; *4*) RoB in measurement of the outcome; and *5*) RoB in selection of the reported result; and overall bias. RoB was assessed for each outcome measured. For each domain, for each outcome, RoB was judged independently by 2 reviewers, as “low,” “high,” or with “some concerns,” based on published information. Criteria for RoB judgments were based on the tool crib sheet [38]. Disagreements between reviewers were resolved by consensus.

### Data synthesis

For this review, the term “article” refers to each individual reference included in the review. The individual pieces of research that are detailed in articles are referred to as “experiments,” and each assessment of aspartame compared with a comparator is referred to as a “study.” The term “comparison” refers to the collection of studies making the same comparison between exposure and comparator. For example, if a parallel-group experiment has 3 arms — 1 arm asked to consume aspartame, 1 arm asked to consume placebo, and 1 arm asked to consume sucrose, this experiment would be considered to consist of 2 studies: aspartame compared with placebo and aspartame compared with sucrose, and these 2 studies contribute to the comparisons between aspartame and placebo, and aspartame and sucrose, respectively. If a parallel-group experiment has 3 arms — 1 arm asked to consume aspartame, 1 arm asked to consume low-dose sucrose, and 1 arm asked to consume high-dose sucrose, this experiment would be considered to consist of 2 studies: aspartame compared with low-dose sucrose and aspartame compared with high-dose sucrose, and these 2 studies would contribute to the comparison between aspartame and sucrose. Thus, an article may detail 1 or more experiments, and each of these may contain 1 or more studies, which may contribute to 1 or more comparisons. Where >1 article reported on the same experiment, additional articles were only included in the review if they provided unique additional information or data relevant to our research questions, with 1 article designated as the “primary reference” for clarity. Articles reporting on subsets of participants, without any variation in study methodology, were not included separately.

All extracted data were tabulated. At the data extraction stage, a number of studies were identified that included an exercise component as well as other relevant aspects. Because exercise may also impact digestive physiology, these studies were not considered beyond this stage unless there was a period before the exercise when the effects of aspartame without exercise had been assessed.

A narrative synthesis of all suitable experiments was subsequently conducted, based on study design type (cross-over/parallel-groups), study duration, aspartame exposure, comparator, and outcomes assessed. Studies were also combined using meta-analysis where ≥10 studies of the same design type and aspartame exposure pattern that investigated the same outcome were available. Only studies of the same design type and aspartame exposure pattern were combined to allow meaningful combination of the data considering the heterogeneity between studies in methodology which may affect study results, and the differing assumptions that may be required based on study design. We considered these characteristics to contribute to the appropriateness of combining study results statistically [43,44]. Furthermore, although only studies of the same exposure pattern were combined, studies were combined regardless of comparator, where comparators were grouped to form subgroups. Meta-analyses were not undertaken where few studies were available with the same design and methodological features to ensure that the combination of studies in this way was meaningful. Data were analyzed as standardized mean difference (SMD) with 95% confidence intervals (CIs), allowing the inclusion of studies regardless of the measure used for the outcome of interest, provided the same measure was used for both intervention and comparator. SMDs were calculated using Hedges’ adjusted g, which includes an adjustment to correct for small sample bias [43,44]. Analyses of cross-over studies also included an adjustment for the correlation between data points from the same individuals in both study arms, assuming a correlation coefficient of *r* = 0.7 [45]. Estimates were calculated using random-effects models primarily due to likely heterogeneity between studies. Fixed-effect models were also applied as sensitivity analyses. Where experiments contributed multiple studies to the same analysis, the number of participants was divided accordingly, such that each participant contributed a maximum of once to each analysis using studies of a parallel-group design, and a maximum of twice to each analysis using studies of a cross-over design. Where experiments provided multiple treatment groups, e.g., lean, overweight, each group was treated as an independent study. Where experiments assessed outcomes multiple times over an extended period, e.g., after 1, 6, and 12 wk, data were used from the longest period over which the intervention remained in place, e.g., at 6 wk after a 6-wk intervention. Where numerical data were not provided, data were extracted from graphs, and multiple outcome assessment time points were used to calculate area under the curve over the period for which comparable data for intervention and comparator were provided. Analyses were conducted using published end-of-intervention mean and SD data. Missing SD data were estimated from the SD data from all other studies using the same measure [46]. Differences, where they occur, between the effects demonstrated in our analyses and those reported in the original articles, will result from the estimations made in our analyses. Heterogeneity between studies was investigated using Higgins JPT et al.’s *I*^2^ statistic [47,48]. Subgroup analyses were undertaken to investigate differing effects as a result of the comparator used. Possible publication bias was investigated using funnel plot asymmetry [43,44]. Additional sources of heterogeneity were not investigated considering the limited number of studies available using comparable methodology. Meta-analyses were undertaken in Stata, version 18 (Stata Corp, Inc).

### Certainty of the evidence

Following our syntheses of the articles found, 2 reviewers (L.R.B. and K.M.A.) also assessed the certainty of the evidence for all primary outcomes, using the GRADE approach [40,41]. The 2 reviewers worked together, to grade the evidence using the criteria: *1*) limitations in study design and implementation; *2*) inconsistency or heterogeneity in the evidence; *3*) indirectness of the evidence; *4*) imprecision in the evidence available; and *5*) other concerns, including risk of publication bias. For each outcome, the certainty of the evidence was assessed as “high,” “moderate,” “low,” or “very low” [40,41].

## Results

### Results of the searches

**FIGURE 1:**
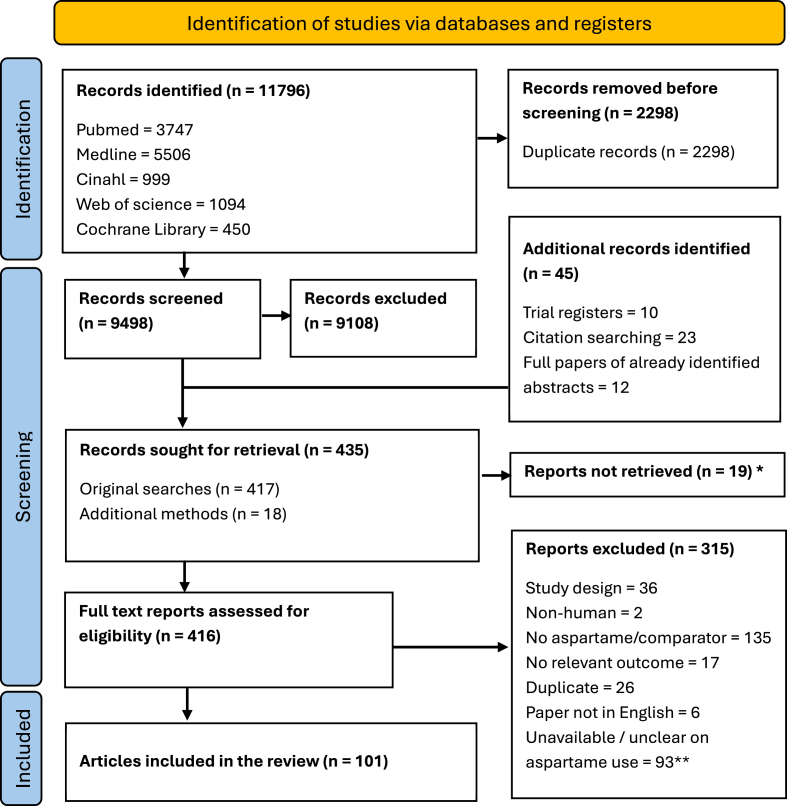
PRISMA diagram. ∗ Includes article record not found (*n* = 7), full study article not available (*n* = 11), and article not retrieved due to copyright (*n* = 1). ∗∗ Where data on aspartame use was unavailable or unclear, clarification was sought from authors. Studies were only included if authors responded by the September 30, 2024, with confirmation of aspartame use. PRISMA, Preferred Reporting Items for Systematic Reviews and Meta-Analyses.

Searches of databases were undertaken on June 10, 2024; all other searches were completed by September 30, 2024. The initial database searches yielded 11,796 search hits, with 9,498 remaining after de-duplication. Following title and abstract screening, 417 articles were considered suitable for full-text screening. Assessment of the trial registries, reference lists, EFSA and FDA libraries, and published abstracts yielded an additional 18 articles for full-text screening, giving 435 articles in total. Following full-text screening, 101 articles were included in the review. The PRISMA flow diagram shows the number of articles at each stage (Figure 1).

### Included studies

The 101 articles included in the review reported on the effects of aspartame, both alone and in conjunction with a number of other substances, compared with those of a number of different comparators, on glucose responses and the actions of a number of appetite-related hormones. Of the 101 articles, 73 articles reported on 77 cross-over experiments [[49], [50], [51], [52], [53], [54], [55], [56], [57], [58], [59], [60], [61], [62], [63], [64], [65], [66], [67], [68], [69], [70], [71], [72], [73], [74], [75], [76], [77], [78], [79], [80], [81], [82], [83], [84], [85], [86], [87], [88], [89], [90], [91], [92], [93], [94], [95], [96], [97], [98], [99], [100], [101], [102], [103], [104], [105], [106], [107], [108], [109], [110], [111], [112], [113], [114], [115], [116], [117], [118], [119], [120], [121]] and 28 articles reported on 23 parallel-groups experiments [[122], [123], [124], [125], [126], [127], [128], [129], [130], [131], [132], [133], [134], [135], [136], [137], [138], [139], [140], [141], [142], [143], [144], [145], [146], [147], [148], [149]]. An overview of all experiments, including all individual studies, is given in Table 1 [[49], [50], [51], [52], [53], [54], [55], [56], [57], [58], [59], [60], [61], [62], [63], [64], [65], [66], [67], [68], [69], [70], [71], [72], [73], [74], [75], [76], [77], [78], [79], [80], [81], [82], [83], [84], [85], [86], [87], [88], [89], [90], [91], [92], [93], [94], [95], [96], [97], [98], [99], [100], [101], [102], [103], [104], [105], [106], [107], [108], [109], [110], [111], [112], [113], [114], [115], [116], [117], [118], [119], [120], [121], [122], [123], [124], [125], [126], [127], [128], [129], [130], [131], [132], [133], [134], [135], [136], [137], [138], [139], [140], [141],[145], [146], [147], [148], [149]]. Detailed study characteristics are given in the excel file in the Supplemental Materials.

**Table 1:** Summary details of all included studies.

Experiment	Focus	Length	Population	N	Aspartame with...	Comparison	Primary outcomes	Secondary outcomes
**Studies with a Cross-over Design**								
Abdallah et al 1997 [49]	Nutrition	<1 day	Lean, nonLCSC	12	Other CHO (polydextrose) (tablet)	Polydextrose (tablet); Sucrose (tablet)	Glucose, Insulin, Glucagon	
Ahmad et al 2020 [50]	Nutrition	2 weeks	Lean, nonLCSC	19	Alone	Sucralose	Glucose, Insulin, GLP-1, Leptin, Fructosamine, HOMA-IR, HOMA- %B, HOMA-%S	
Akalp et al 2023 [51]	Exercise & Nutrition	<1 day	Trained, Lean	10	Alone	Taurine	Glucose	
Ali et al 2016 [52]	Exercise & Nutrition	<1 day	Trained	10	Alone (encapsulated)	Caffeine (encapsulated)	Glucose, Insulin	
Anton et al 2010 [53]	Nutrition	<1 day	Lean; Obese	19; 12	Nutritive	Stevia & nutritive; Sucrose & nutritive	Glucose, Insulin	Energy intake; Appetite - hunger; satiety; fullness; Adverse events
Berlin et al 2005 [54]	Nutrition	<1 day	Healthy	12	Alone	Glucose 32.5g; Glucose 75g	Glucose, Insulin	
Bird et al 2013 [55]	Exercise & Nutrition	<1 day	Trained	21	Alone	Multi-nutrient supplement	Glucose	
Bonnet et al 2018 [56]	Nutrition	12 weeks	Lean or Overweight, LCSC	60	Acesulfame K	Water	Glucose, Insulin, Matsuda Index, HOMA-IR, Insulinogenic Index, Disposition Index, Stumvoll Indices	Energy intake
Bryant et al 2014 [57]	Nutrition	<1 day	Healthy	10	Glucose	Glucose; Glucose & Acesulfame K; Glucose & saccharin	Glucose	Appetite - hunger; fullness
Bruce et al 1987 [58] Experiment 2	Nutrition	<1 day	Lean	7	Alone; with dextrose	Unflavoured gum & water vehicles; with dextrose	Glucose, Insulin	
Bruce et al 1987 [58] Experiment 3	Nutrition	<1 day	Lean	5	Alone	Water	Glucose, Insulin	
Burns et al 1991 [59]	Nutrition	<1 day	Healthy	8	Alone; Sucrose	Unsweetened beverage; Sucrose	Glucose, Insulin, Glucagon	
Carlson and Shah 1988 [60]	Nutrition	<1 day	Healthy	16	Alone (encapsulated); Alone (rinse); Alone (drink)	Aspartic acid (encapsulated); Phenylalanine 0.3g (encapsulated); Phenylalanine 1.0g (encapsulated)	Glucose, Insulin	
Chong et al 2014 [61]	Exercise & Nutrition	<1 day	Trained	12	Alone	Glucose; Maltodextrin; Water	Glucose	
Chryssanthopou-los et al 2008 [62]	Exercise & Nutrition^1^	<1 day	Healthy	8	Alone (single dose)	High CHO meal (single dose); High CHO meal (multiple doses)	Glucose, Insulin	Appetite - fullness
Coggan and Coyle 1989 [63]	Exercise & Nutrition	<1 day	Trained	6	Alone	Glucose & sucrose	Glucose, Insulin	
Colagiuri et al 1989 [64]	Nutrition	6 weeks	NIDDM	9	Nutritive	Sucrose & nutritive	Glucose, Insulin, HbA1c	
Cuomo et al 2011 [65]	Nutrition	<1 day	Healthy	10	Acesulfame K (non-carbonated) & variable nutritive (solid); Acesulfame K (non-carbonated) & variable nutritive (liquid); Acesulfame K (carbonated) & variable nutritive (solid); Acesulfame K (carbonated) & variable nutritive (liquid)	Water & variable nutritive (solid); Water & variable nutritive (liquid);	Glucose, Ghrelin, Cholecystokinin	Energy intake; Appetite - hunger; satiety; desire to eat; prospective consumption; Adverse events
Fahey et al 1991 [66]	Exercise & Nutrition	<1 day	Trained	5	Alone	Polylactate (sodium lactate); Glucose polymer (maltodextrin)	Glucose	
Finassi et al 2023 [67]	Nutrition	<1 day	Healthy	15	Acesulfame K & Na cyclamate in diet drink; Acesulfame K & Na cyclamate in water;	Sucrose in regular drink; Sucrose in water; water	Insulin	
Fukuda et al 2010 [68]	Nutrition	<1 day	Mild untreated DM	38	Acesulfame K & erythritol & nutritive - meal; sweets	Sucrose & nutritive vehicle - meal; sweets;	Glucose, Insulin	Adverse events
Gam et al 2014 [69]	Exercise & Nutrition	<1 day	Trained	14	Alone	Quinine; Water; Nothing	Glucose	
Green et al 2001 [70]	Nutrition	<1 day	Healthy	26	Alone & told placebo; Alone & told glucose	Glucose & told placebo; Glucose & told glucose	Glucose	
Hall et al 2003 [71]	Nutrition	<1 day	Healthy	6	Alone (encapsulated); & with Nutritive	l-aspartic acid and l-phenylalanine (encapsulated); & with nutritive; Corn flour (encapsulated); & with nutritive	Glucose, Insulin, GLP-1, GIP, Cholecystokinin	Appetite - hunger, desire to eat; fullness
Hargreaves & Briggs 1988 [72]	Exercise & Nutrition	<1 day	Trained	5	Alone	Glucose polymer (Polycose)	Glucose, Insulin	
Horwitz et al 1988 [73]	Nutrition	<1 day	Lean; NIDDM	12; 10	Alone (diet drink)	Saccharin (diet drink); Diet drink	Glucose, Insulin, Glucagon	Adverse events
Karamanolis et al 2011 [74]	Exercise & Nutrition^1^	<1 day	Trained	9	Alone	Nutritive (low GI); Nutritive (high GI)	Glucose, Insulin	
Kashima et al 2019 [75]	Nutrition	<1 day	Healthy	9	Alone after water preload; Alone after Gymnema Sylvestre	Glucose after water preload; Glucose after Gymnema Sylvestre	Glucose, Insulin	
Kim et al 2020 [76]	Nutrition	2 weeks	Healthy	50	Acesulfame K	Water	Glucose, Insulin, HOMA-IR, Stumvoll Index, Matsuda index	
Kimura et al 2017 [77]	Nutrition	<1 day	Lean	13	Alone; with Nutritive	d-allulose; with Nutritive	Glucose, Insulin	
Kingwell et al 1989 [78]	Exercise & Nutrition	<1 day	Trained, Lean	9	Alone	Glucose polymer (Polycose)	Glucose	
Koch et al 2001 [79]	Exercise & Nutrition	<1 day	Trained	10	Alone	Carbohydrate (maltodextrin/ dextrose) solution (Gatorlode)	Glucose	
Kumar et al 2019 [80]	Exercise & Nutrition	<1 day	Trained; Healthy	12; 12	Nutritive	Caffeine & Nutritive; CHO & Nutritive; Caffeine+CHO & Nutritive	Glucose	
Lapierre et al 1990 [81]	Nutrition	<1 day	Healthy	14	Alone (encapsulated) & Nutritive	Placebo (encapsulated) & nutritive	Glucose	Appetite – hunger; Adverse events
Lehmann et al 2021 [82]	Nutrition	<1 day	Post-bariatric Hypo-glycaemia	12	Alone	Glucose	Glucose	Adverse events – hypoglycaemia symptoms
Maersk, Belza, Holst, et al 2012 [83]	Nutrition	<1 day	Overweight or Obesity	24	Alone (diet cola)	Regular cola; Milk; Water	Glucose, Insulin, Ghrelin, GLP-1,GIP	Energy intake; Appetite - hunger; fullness; prospective consumption; thirst
Melanson et al 1999 [84]	Nutrition	<1 day	Healthy	10	Alone with variable nutritive	CHO drink with variable nutritive; High fat drink with variable nutritive	Glucose	Energy intake; Appetite - hunger; satiety; desire to eat
Melchior et al 1991 [85]	Nutrition	<1 day	Lean	10	Nutritive	Nothing, Nutritive vehicle + Sucrose	Glucose, Insulin	Appetite; hunger
Millard-Stafford et al 1992 [86]	Exercise & Nutrition^1^	<1 day	Trained; Lean	8	Alone	Glucose polymers / fructose / electrolyte drink	Glucose	
Moller 1991 [87]	Nutrition	<1 day	Healthy	6	Alone	Water; Bovine albumin in water	Glucose, Insulin	
Nassis et al 1998 [88]	Exercise & Nutrition	<1 day	Trained	9	Alone	Carbohydrate – electrolyte drink (Lucozade Sport)	Glucose	
Nguyen et al 1998 [89]	Nutrition	<1 day	Lean	7	Alone	Glucose	Glucose, Insulin	
Noriega et al 1997 [90] Experiment 2	Exercise & Nutrition^1^	<1 day	Lean	6	Alone	Rice; Bread	Glucose, Insulin	
Okuno et al 1986 [91] Single administration	Nutrition	<1 day	Healthy; untreated DM of differing degrees)	7; 22	Alone	Glucose	Glucose, Insulin, Glucagon	
Okuno et al 1986 [91] Continuous administration	Nutrition	2 weeks	Untreated DM	9	Alone	Glucose	Glucose	
Osterberg et al 1985 [92]	Exercise & Nutrition	<1 day	Trained	15	Alone; Alone & electrolytes	3% CHO & electrolytes; 6% CHO & electrolytes; 12% CHO & electrolytes;	Glucose, Insulin	
Panahi et al 2013 [93]	Nutrition	<1 day	Lean	32	Variable Nutritive (diet cola)	Water & variable Nutritive, Milk & variable Nutritive, Orange juice & variable Nutritive, Regular cola & variable Nutritive	Glucose	Energy intake; Appetite - thirst; motivation to eat; desire to eat; hunger; fullness; prospective consumption.
Pearson et al 2023 [94]	Nutrition	<1 day	Healthy	8	Nutritive (diet cola)	Regular cola & Nutritive; Water & Nutritive	Glucose, Insulin	Appetite - hunger; thirst; desire to eat; nausea; amount you could eat
Prat-Larquemin et al 2000 [95]	Nutrition	<1 day	Lean	24	Maltodextrin & Nutritive	Maltodextrin & nutritive; Sucrose & nutritive	Glucose, Insulin	Appetite - hunger
Preechasuk et al 2023 [96]	Nutrition	12 weeks	NIDDM	16	Alone	Allulose	Glucose, Insulin, HOMA-IR, HbA1c, GLP-1, GIP, HOMA-B, Matsuda Index, Insulinogenic Index	Adverse events
Rodin 1990 [97]	Nutrition	<1 day	Lean; Overweight	12; 12	Alone (lemon-flavoured)	Fructose (lemon-flavoured); Glucose (lemon-flavoured); Water	Glucose, Insulin, Glucagon	Energy intake
Sathyapalan et al 2015 [98]	Nutrition	<1 day	Aspartame sensitive; non-sensitive	53; 49	Nutritive	Nutritive vehicle	Glucose, Insulin, HOMA-IR, GLP-1, GIP	Appetite – hunger; thirst; Adverse events
Schiffman et al 1987 [99]	Nutrition	<1 day	Aspartame sensitive	40	Alone (encapsulated)	Cellulose placebo (encapsulated)	Glucose, Insulin, Glucagon	Adverse events
Shigeta et al 1985 [100] Experiment 2a	Nutrition	<1 day	NIDDM	15	Alone	Glucose	Glucose, Insulin	
Short et al 1997 [101]	Exercise & Nutrition^1^	<1 day	Trained	8	Alone	22.5g CHO (maltodextrin & dextrose); 45g CHO; 75g CHO	Glucose, Insulin	
Siegler et al 2012 [102]	Exercise & Nutrition^1^	<1 day	Lean	9	Maltodextrin (A); Maltodextrin & sucrose (CA)	Maltodextrin & sucrose (C); Water	Glucose, Insulin	
Singleton et al 1999 [103]	Nutrition	<1 day	Healthy	22	Nutritive (Dairy)	Dairy vehicle, Dairy vehicle & fructose; Dairy vehicle & glucose	Glucose, Insulin	
Smeets et al 2005 [104]	Nutrition	<1 day	Lean	5	Alone	Glucose; Maltodextrin; Water	Glucose, Insulin	
Soenen and Westerterp-Plantenga 2007 [105] Experiment 1	Nutrition	<1 day	Healthy	30	Acesulfame K & sodium cyclamate	Sucrose; HFCS; Milk	Glucose, Insulin, GLP-1, Ghrelin	Appetite - hunger; satiety; fullness; desire to eat; prospective consumption
Solomi et al 2019 [106]	Nutrition	<1 day	Lean or Overweight	10	Acesulfame K (diet cola) & glucose	Glucose (water); Sucrose (regular cola)	Glucose	
Sorrentino et al 2020 [107]	Nutrition	<1 day	Lean	12	Alone	Erythritol	Ghrelin	Appetite - hunger; satisfied; fullness; desire to eat; desire for sweet; desire for salt; desire for savoury; desire for fatty
Spiers et al 1998 [108]	Nutrition	20 days	Healthy	48	Alone (soda & encapsulated) - High dose (45mg/kg BW/d) or Low dose (15mg/kg BW/d)	Sucrose (soda & encapsulated); Placebo (unsweetened soda & cellulose & silicon dioxide capsules)	Glucose, Insulin	Adverse events
Stannard et al 2000 [109]	Exercise & Nutrition^1^	<1 day	Trained	10	Alone (diet drink)	Glucose (water); Food item	Glucose	
Steinert et al 2011 [110] Full Study	Nutrition	<1 day	Lean	12	Alone (intragastric)	Acesulfame K (intragastric); Sucralose (intragastric); Fructose (intragastric); Glucose (intragastric); Water (intragastric)	Glucose, Insulin, GLP-1, Ghrelin, PYY, Glucagon	Appetite - hunger; satiety; fullness; Adverse events
Sturm et al 2004 [111]	Nutrition	<1 day	Young; Older	12; 12	Nutritive (250 kcal yoghurt drink)	Nutritive (750 kcal yoghurt drink); Water	Glucose, Insulin, Cholecystokinin	Energy intake, Appetite - hunger; fullness
Sylvetsky et al 2016 [112] Study Arm 2	Nutrition	<1 day	Healthy	31	Sucralose (18 mg) & acesulfame-K (18mg) in diet drink & Glucose	Sucralose (68mg) & acesulfame-K (41mg) in diet drink & Glucose; Sucralose (68mg) & acesulfame-K (41mg) in seltzer water & Glucose; Seltzer water & Glucose	Glucose, Insulin, GLP-1, GIP	Appetite - hunger; satiety
Tamis-Jortberg et al 1996 [113]	Exercise & Nutrition	<1 day	DM, NIDDM	25	Alone & electrolytes	Glucose polymers, fructose & electrolytes	Glucose, Insulin	
Teff 2010 [114] Experiment 3	Nutrition	<1 day	Lean	12	1g dose & nutritive (tasted, not ingested); 20g dose & nutritive (tasted, not ingested)	0.6g salt & nutritive (tasted, not ingested), 6g salt & nutritive (tasted, not ingested), Nothing	Glucose, Insulin, Pancreatic polypeptide	
Teff et al 1995 [115] Experiment 1	Nutrition	<1 day	LCSC	15	Alone (tasted, not ingested, 1min exposure)	Water; Saccharin; Sucrose; Food item (all tasted, not ingested, 1min exposure)	Glucose, Insulin	
Teff et al 1995 [115] Experiment 2	Nutrition	<1 day	Healthy	16	Alone (tasted, not ingested, 3min exposure)	Water; Saccharin; Sucrose; Food item (all tasted, not ingested, 3min exposure)	Glucose, Insulin	
Temizkan et al 2015 [116]	Nutrition	<1 day	Healthy; NIDDM	8; 8	Glucose	Sucralose & Glucose; Water & Glucose	Glucose, Insulin, GLP-1	
Tey et al 2017 [117]	Nutrition	<1 day	Lean	31	Alone; with variable nutritive	Stevia; Sucrose; Monk fruit; all with variable nutritive	Glucose, Insulin	Energy intake; Appetite - hunger; desire to eat; fullness; prospective consumption
Warwick et al 1993 [118]	Nutrition	<1 day	Lean	15	Tasty high CHO food item; Tasty high fat food item	Bland high CHO food item; Bland high fat food item	Glucose	Energy intake; Appetite - hunger; fullness
Wax et al 2013 [119]	Exercise & Nutrition^1^	<1 day	Trained	6	Saccharin	CHO	Glucose	
Wolf-Novak et al 1990 [120]	Nutrition	<1 day	Healthy; PKU	7; 7	Alone; CHO beverage	Unsweetened vehicle; CHO beverage vehicle	Glucose, Insulin	
Wouassi et al 1997 [121]	Exercise & Nutrition^1^	<1 day	Healthy	7	Alone	Glucose	Glucose, Insulin, Glucagon	
**Studies with a Parallel-groups Design**								
Benton & Owens 1993 [122] Experiment 1	Nutrition	<1 day	Healthy	153	Acesulfame K	Glucose	Glucose	
Benton & Owens 1993 [122] Experiment 2	Nutrition	<1 day	Healthy	53	Acesulfame K	Glucose	Glucose	
Ebbeling et al 2020 [123]	Nutrition	12 months	Lean, Overweight or Obesity	203	Other LCS (Diet drinks)	Sugar-sweetened drinks; Water	Glucose, Insulin, HOMA-%B, HOMA-%S	Energy intake; Adverse events
Engel et al 2018 [124, (125,135)]	Nutrition	6 months	Overweight or Obesity	73	Alone (Diet cola)	Sucrose-sweetened cola; Water; Milk	Glucose, Insulin (fasting, OGTT), HOMA-IR, Matsuda Index, Leptin	Energy Intake
Finley et al 2019 [126]	Nutrition	<1 day	Healthy	371	Alone ingested	Glucose ingested; Glucose tasted, but not ingested	Glucose	
Gozal et al 1985 [127]	Exercise & Nutrition	<1 day	Trained	26	Alone orally	Glucose intravenously; Glucose orally	Glucose, Insulin, Glucagon	
Harrold et al 2024 [128]	Nutrition	52 weeks	Overweight or Obesity, LCSC	493	Other LCS (Diet drinks)	Water	Glucose, Insulin, HbA1c	Appetite - hunger
Hieronimus et al 2024 [130, (129,142,143)]	Nutrition	16 days	Lean, Overweight or Obesity	187	Alone; 10%Ereq High Fructose Corn Syrup (HFCS)	17.5%Ereq HFCS; 17.5%Ereq Fructose; 25%Ereq HFCS; 25%Ereq Fructose; 25%Ereq Glucose; 25%Ereq Sucrose	Glucose, Insulin (fasting; 24hr; OGTT, amplitudes), HOMA-IR Matsuda Index, Predicted M Index, Stumvoll Index, Surrogate hepatic IR Index, Leptin	Energy intake
Higgins et al 2018 [131]	Nutrition	12 weeks	Lean, non-LCSC	100	Dextrose + 350mg dose; Dextrose + 1050mg dose (some encapsulated)	Dextrose vehicle	Glucose, Insulin (fasting, OGTT), HbA1c, GLP-1, GIP, Leptin	Appetite - hunger; desire to eat; thirst; prospective consumption; fullness; preoccupation with food
Higgins and Mattes 2019 [132]	Nutrition	12 weeks	Overweight or Obesity, non-LCSC	154	Alone	Sucrose; Saccharin; Rebaudioside A; Sucralose	Glucose, Insulin (fasting, OGTT), HbA1c	Energy intake; Appetite - hunger; fullness; desire to eat; prospective consumption; thirst; preoccupation with food
Kendig et al 2023 [133]	Nutrition	12 weeks	Lean or Overweight	118	Acesulfame K & Sucralose (Diet Soda)	Water; Sucrose-sweetened soda	Glucose (OGTT)	
Knopp et al 1976 [134]	Nutrition	13 weeks	Overweight	59	Alone (encapsulated)	Lactose (encapsulated)	Glucose, Insulin, Glucagon	Adverse events
Markus and Rogers 2020 [136] Experiment 1	Nutrition	<1 day	Healthy	90	Alone	Sucrose; Milk	Glucose	Appetite – hunger; fullness; desire to eat a meal; desire to eat a snack; Adverse events – hypoglycaemia symptoms
Martin and Benton 1999 [137]	Nutrition	<1 day	Healthy	80	Saccharin; Saccharin & nutritive	Glucose; Glucose & nutritive	Glucose	
Nehrling et al 1985 [138]	Nutrition	18 weeks	NIDDM & IDDM	62	Alone (encapsulated)	Corn starch (encapsulated)	Glucose, Glycated Haemoglobin (HbA1c)	Adverse events
Orku et al 2023 [139]	Nutrition	4 weeks	Lean, non-LCSC	48	Acesulfame K	Saccharin; Sucralose; Water	Glucose, Insulin (fasting; OGTT), HOMA-IR, HbA1c, GLP-1, Matsuda Index	Energy intake
Peters et al 2016 [140]	Nutrition	52 weeks	Overweight or Obesity, LCSC	303	Other LCS	Water	Glucose (fasting)	Appetite - hunger
Sorensen et al 2005 [144,(141)]	Nutrition	10 weeks	Overweight	41	Cyclamate, Acesulfame K & Saccharin + nutritive (foods and drinks)	Sucrose + nutritive (foods and drinks)	Glucose, Insulin, HOMA-IR	Energy intake; Appetite - hunger; fullness; diurnal
Suez et al 2022 [145]	Nutrition	14 days	Lean or Overweight, non-LCSC	131	Glucose	Saccharin & glucose; Sucralose & glucose; Stevia & glucose; Glucose Alone; Nothing	Glucose, Insulin (OGTT), CGM (CoV)), HbA1c, GLP-1	Energy intake
Sunram-Lea et al 2001 [146]	Nutrition	<1 day	Lean or Overweight	60	Alone fasting; Alone 2hr post breakfast; Alone 2hr post lunch	Glucose fasting; Glucose 2hr post breakfast; Glucose 2hr post lunch	Glucose	
Sunram-Lea et al 2004 [147]	Nutrition	<1 day	Healthy	40	Nutritive fat-free; Nutritive full-fat	Glucose & nutritive fat-free; Glucose & nutritive full fat	Glucose	
Virkkunen et al 1994 [148]	Nutrition	<1 day	Healthy; Antisocial disorder; Explosive disorder; non-impulsive disorders	79	Alone	Glucose	Glucose, Insulin, Glucagon	
Wise et al 1989 [149]	Nutrition	5 days	IDDM, Lean	16	Nutritive	Sucrose & nutritive	Glucose, Fructosamine	

Type: Nutrition - the focus of the study was on nutritional aspects; Exercise & Nutrition – the study aims to explore the impact of exercise and nutrition;

1 study involving exercise, but with some readings pre-exercise unaffected by exercise; Length: lasting 1 day or less, classified for analyses as acute; 2 - 30 days, classified for analyses as medium-term; > 30 days, classified for analyses as long-term; Population: Body weight, usual LCS use, diabetes, PKU are given if stipulated as inclusion criteria, all participants were otherwise healthy; LCSC: low-calorie-sweetener consumer; non-LCSC: non-, rare or irregular low-calorie-sweetener consumer; DM: diabetes mellitus; IDDM: insulin-dependent diabetes mellitus; NIDDM: non-insulin-dependent diabetes mellitus; PKU: phenylketonuria; Aspartame with …: Alone - refers to zero kcal delivery; Other LCS – specified if given; Sugars – specified if given; Nutritive - delivery includes calories, e.g. as part of a milkshake, food item; CHO – carbohydrate; Outcomes – CCK: cholesystokinin; GLP-1: glucagon like peptide-1; GIP: glucose dependent insulinotropic peptide; HbA1c: Haemoglobin A1C (average blood glucose measures over the past 2-3 months); HOMA-%B: Homeostatic Model Assessment for Beta-cell function; HOMA-IR: Homeostatic Model Assessment for Insulin Resistance; HOMA-%S: Homeostatic Model Assessment for Insulin Sensitivity; OGTT: Oral Glucose Tolerance Test; Predicted M Index: PYY: Polypeptide Tyrosine Tyrosine; CGM (CoV): Continuous Glucose Monitoring (Coefficients of Variance).

#### Cross-over studies

The 77 cross-over experiments included 23 experiments, which involved an exercise component, and although 9 of these also included a pre-exercise rest period where data of relevance to our research questions could be gained [62,74,86,90,101,102,108,119,121], 14 of these experiments only measured digestive physiology during or after exercise and were not considered further [51,52,55,61,63,66,69,72,[78], [79], [80],^88^,^92^,^113^]. Of the 54 nutritional experiments, 12 experiments involved individuals or a subgroup of individuals where physiology in relation to aspartame or digestion may be compromised or unusual. These experiments involved individuals with aspartame sensitivity (2 experiments [98,99]), phenylketonuria (PKU) (1 experiment [120]), untreated diabetes mellitus (DM) (3 experiments [68,91]), non–insulin-dependent diabetes mellitus (NIDDM) (5 experiments [64,73,96,100,116]), and 1 experiment involved individuals with postbariatric hypoglycemia [82]. These experiments or subgroups of individuals were considered separately. Where experiments included subgroups of individuals without compromise [73,91,98,116,120], these subgroups were considered with all other studies on healthy adults.

##### Acute studies

With the aforementioned considerations, 55 experiments lasted for 1 d or less in duration and could provide data that were unaffected by exercise, and 51 of these experiments involved healthy adults or a healthy adult subgroup. Provision of aspartame, comparator/s, and outcomes investigated in these studies is given in Supplemental Table 1. A wide range was found in aspartame provision and comparator/s used. Thirty-eight studies investigated aspartame when provided alone and compared this with effects from vehicle (7 studies [58,59,73,87,104,120]), vehicle plus glucose (10 studies [54,70,75,89,91,97,104,121]), sucrose (3 studies [59,83,117]), fructose (1 study [97]), and glucose and fructose (1 study [86]), vehicle plus non–sweet-tasting carbohydrates (4 studies [101,104]), vehicle plus other nutritive components (7 studies [62,74,87,90,120]), or 5 different LCS (5 studies [73,77,107,117]). Eight studies investigated aspartame in combination with other LCS, both with and without other nutritive components, and compared these with effects from vehicle (1 study [67]), vehicle plus sucrose (3 studies [67,105]), high fructose corn syrup (1 study [105]), carbohydrate (1 study [119]), vehicle plus additional LCS (1 study [112]), or sucrose (1 study [106]). Twenty-three studies investigated aspartame in combination with sugars or other nutritive components, e.g., in a milkshake, food item, as part of a meal, or in the form of an oral glucose tolerance test, and compared these with effects from vehicle (10 studies [49,[57], [58], [59],^95^,^98^,^102^,^103^,^116^,^120^]), vehicle plus 5 different LCS (5 studies [53,57,77,116]), vehicle plus sugars (6 studies [53,85,94,102,103]), and sugars (2 studies [49,95]). Fourteen studies also investigated aspartame without taste, almost all with a different comparator [60,71,75,81,110]), and 10 studies investigated the effects of aspartame without ingestion, again with a variety of comparators [114,115].

The majority of studies assessed responses in glucose and insulin, using a number of different methods, with very few studies also investigating other appetite-regulating hormones. With ≥10 studies available, meta-analyses were analyzed to investigate the effects of aspartame when provided alone and in combination with nutritive components on glucose and insulin responses.

###### Meta-analysis 1. (aspartame alone, glucose responses)

**FIGURE 2:**
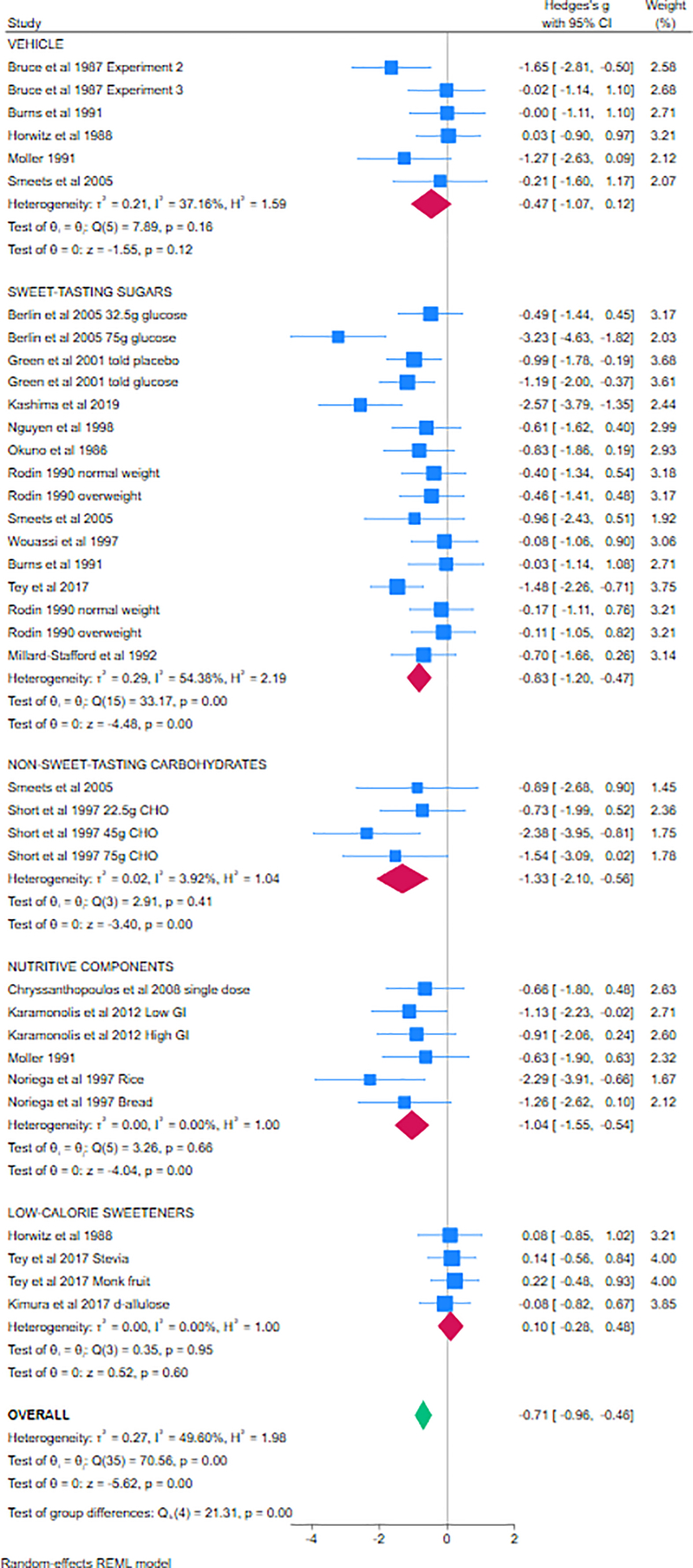
Forest plot for meta-analysis 1 investigating the effects of aspartame administered alone on glucose responses in healthy individuals (cross-over studies). Individual studies are represented by the blue boxes; combined effects are represented by the diamonds. The *x*-axis demonstrates the effect size, in standard deviations, as calculated using Hedges’ adjusted *g*. Studies on the 0 line demonstrate no differences between aspartame and comparator, studies to the right of the 0 line demonstrate greater responses to aspartame, studies to the left of the 0 line demonstrate reduced responses to aspartame / increased responses to comparator. CHO, carbohydrate. GI: Glycaemic Index; REML: restricted Maximum Likelihood.

Thirty-four studies provided aspartame alone and were divided into 5 subgroups dependent on comparator (vehicle, sweet-tasting sugars, non–sweet-tasting carbohydrates, nutritive components, and other LCS); suitable data were not available for inclusion from 4 studies [83,107,120]. Using random-effects models, no effects of aspartame were found when compared with vehicle (SMD = −0.47; 95% CI: −1.07, 0.12; *I*^2^ = 37%, 6 studies) or when compared with other LCS (SMD = 0.10; 95% CI: −0.28, 0.48; *I*^2^ = 0%, 4 studies), but significantly lower levels of blood glucose were found following aspartame when compared with sweet-tasting sugars (SMD = −0.83; 95% CI: −1.20, −0.47; *I*^2^ = 54%, 14 studies [2 with 2 population subgroups]), non–sweet-tasting carbohydrates (SMD = −1.33; 95% CI: −2.10, −0.56; *I*^2^ = 4%, 4 studies), and other nutritive components (SMD = −1.04; 95% CI: −1.55, −0.54; *I*^2^ = 0%, 6 studies). The Forest plot is given in Figure 2. Statistically significant differences were found between subgroups (*χ*^2^ = 21.31; *P* < 0.01). The overall effect estimate (SMD = −0.71; 95% CI: −0.96, −0.46; *I*^*2*^ = 50%, 34 studies) reflects the subgroups and studies included. Fixed-effects models revealed similar effects (Supplemental Materials), as do narrative reports from the studies not included in the analyses. The funnel plot revealed some asymmetry, suggestive of publication bias (Supplemental Figure 1).

###### Meta-analysis 2 (aspartame with a nutritive component, glucose responses)

Nineteen studies provided aspartame with a nutritive element and were divided into 4 subgroups dependent on comparator (nutritive vehicle, nutritive vehicle with LCS, nutritive vehicle with nutritive sugars, and sugars); suitable data were not available for blood glucose for inclusion from 4 studies [58,103]. Using random-effects models, no effects of aspartame were found when compared with nutritive vehicle (SMD = 0.01; 95% CI: −0.26, 0.27; *I*^2^ = 0%, 8 studies), nutritive vehicle with LCS (SMD = 0.22; 95% CI: −0.30, 0.74; *I*^2^ = 35%, 5 studies), nutritive vehicle and sugars (SMD = −0.40; 95% CI: −0.93, 0.13; *I*^2^ = 34%, 4 studies), or when compared with sugars (SMD = −0.04; 95% CI: −1.05, 0.97; *I*^2^ = 66%, 2 studies). The overall effect estimate (SMD = −0.02; 95% CI: −0.22, 0.18; *I*^2^ = 9%, 19 studies) also demonstrated no effects, with no statistically significant differences between subgroups (*χ*^2^ = 2.81; *P* = 0.42). The Forest plot is given in Supplemental Figure 2. Fixed-effects models also revealed similar effects (Supplemental Materials), as do narrative reports from the studies not included in the meta-analysis. The funnel plot reveals limited asymmetry, suggestive of little publication bias (Supplemental Figure 3).

###### Meta-analysis 3 (aspartame alone, insulin responses)

**FIGURE 3:**
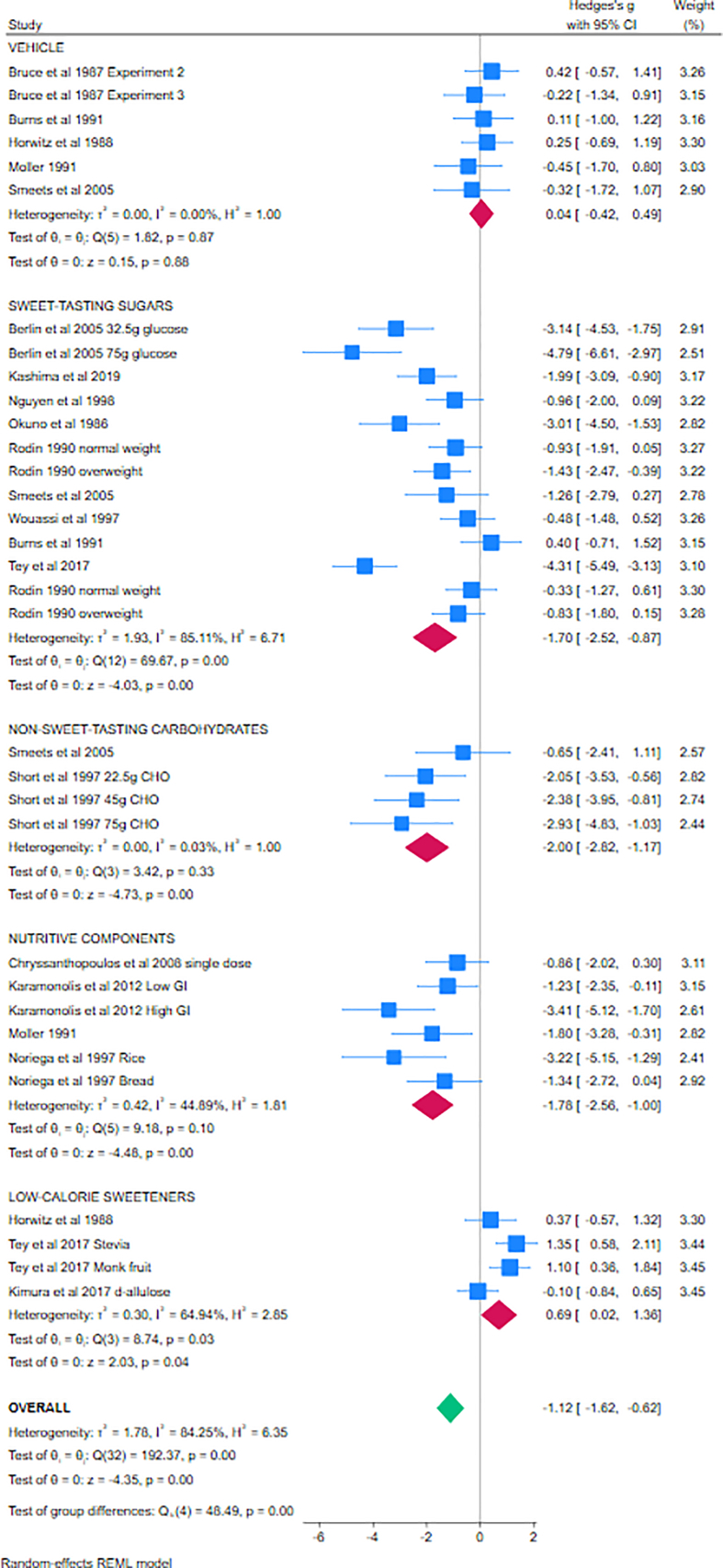
Forest plot for meta-analysis 3 investigating the effects of aspartame administered alone on insulin responses in healthy individuals (cross-over studies). Individual studies are represented by the blue boxes; combined effects are represented by the diamonds. The *x*-axis demonstrates the effect size, in standard deviations, as calculated using Hedges’ adjusted *g*. Studies on the 0 line demonstrate no differences between aspartame and comparator, studies to the right of the 0 line demonstrate greater responses to aspartame, studies to the left of the 0 line demonstrate reduced responses to aspartame / increased responses to comparator. CHO, carbohydrate. GI: Glycaemic Index; REML: restricted Maximum Likelihood.

For insulin responses, meta-analysis 3 included 31 studies, all providing aspartame alone, divided into 5 subgroups dependent on comparator (vehicle, sweet-tasting sugars, non–sweet-tasting carbohydrates, nutritive components, and other LCS); suitable data were not available for inclusion from 7 studies [70,83,85,107,120]. Using random-effects models, no effects of aspartame were found when compared with vehicle (SMD = 0.04; 95% CI: −0.42, 0.49; *I*^2^ = 0%, 6 studies); significantly lower levels of blood insulin were found following aspartame when compared with sweet-tasting sugars (SMD = −1.70; 95% CI: −2.52, −0.87; *I*^2^ = 85%, 11 studies), non–sweet-tasting carbohydrates (SMD = −2.00; 95% CI: −2.82, −1.17; *I*^2^ = 0%, 4 studies), or other nutritive components (SMD = −1.78; 95% CI: −2.56, 1.00, *I*^2^ = 45%, 6 studies); and slightly higher levels of blood insulin were found following aspartame when compared with other LCS (SMD = 0.69; 95% CI: 0.02, 1.36; *I*^2^ = 65%, 4 studies). The differences between subgroups were statistically significant (*χ*^2^ = 48.49; *P* < 0.01). The Forest plot is given in Figure 3. The overall effect estimate (SMD = −1.12; 95% CI: −1.62, −0.62; *I*^2^ = 84%, 31 studies) reflects the subgroups and studies involved. Fixed-effects models revealed similar effects (Supplemental Materials), as do narrative reports from the studies not included in the meta-analysis. The funnel plot revealed some asymmetry, suggestive of publication bias (Supplemental Figure 4).

###### Meta-analysis 4 (aspartame with a nutritive component, insulin responses)

Sixteen studies provided aspartame with a nutritive element and were divided into 4 subgroups dependent on comparator (nutritive vehicle, nutritive vehicle with LCS, nutritive vehicle with nutritive sugars, and sugars); suitable data were not available for inclusion from 7 studies [57,58,103]. Using random-effects models, no effects of aspartame were found when compared with nutritive vehicle (SMD = 0.03; 95% CI: −0.24, 0.30; *I*^2^ = 0%, 7 studies), nutritive vehicle and LCS (SMD = −0.03; 95% CI: −0.46, 0.40; *I*^2^ = 0%, 3 studies), or sugars (SMD = 0.11; 95% CI: −0.98, 1.21; *I*^2^ = 71%, 2 studies), and a lower blood insulin following aspartame was found when compared with nutritive vehicle and sugars (SMD = −0.51; 95% CI: −0.92, −0.10; *I*^2^ = 0%, 4 studies). The overall effect estimate (SMD = −0.07; 95% CI: −0.26, 0.12; *I*^2^ = 0%, 16 studies) demonstrates no effects, and there were no statistically significant differences between subgroups (*χ*^2^ = 4.90; *P* = 0.18). The Forest plot is given in Supplemental Figure 5. Fixed-effects models revealed similar effects (Supplemental Materials) as do narrative reports from the studies not included in the meta-analysis. The funnel plot revealed little asymmetry, suggestive of little publication bias (Supplemental Figure 6).

Where other appetite-regulating hormones were assessed, few effects were found. A few studies also assessed energy intake, appetite, and/or adverse events. Where energy intake and appetite were assessed, effects mirrored those in blood glucose and insulin — no differences were found when aspartame was compared with placebo or other LCS, but significant reductions were found when aspartame was compared with sugars or other nutritive compounds. Few adverse events were reported across all studies.

Three acute experiments involved individuals with sensitivity to aspartame [98,99,120]. In one experiment, participants who self-reported aspartame sensitivity demonstrated no effects of aspartame in a cereal bar on fasting glucose, insulin, insulin sensitivity, or adverse events, although an increase in glucagon-like-peptide-1 (GLP-1) and a decrease in glucose-dependent insulinotropic peptide (GIP) were found, when compared with an aspartame-free cereal bar vehicle [98]. In a second experiment [99], blood glucose was reported to be lower at one time point following encapsulated aspartame compared with placebo, but no data or discussion of this finding are given. There were no differences at other time points, and no effects in plasma insulin, glucagon, or adverse events were reported. In one experiment with adolescents with PKU [120], no effects of aspartame were again reported, although a carbohydrate load (with and without aspartame) increased plasma glucose and insulin.

Five acute experiments involved individuals with some degree of DM [68,73,91,100,116]. In these experiments, no effects of aspartame were found when compared with vehicle or saccharin, for blood glucose, insulin, glucagon, or for adverse events [73]. When compared with glucose, aspartame resulted in lower plasma glucose and insulin in one study [100], and lower blood glucose but no effects in insulin or glucagon in another study [91]. Aspartame in combination with acesulfame K, erythritol, and nutritive elements resulted in lower blood glucose and insulin and similar adverse events compared with the same nutritive elements and sucrose [68]. When combined with glucose, aspartame had no effects on plasma glucose, insulin, or GLP-1 when compared with vehicle or sucralose [116].

The experiment in individuals with postbariatric hypoglycemia [82] also reported no effects of aspartame on blood glucose, and no adverse events, but the expected increases and decreases in response to glucose in this population were found. In all these experiments, in relation to aspartame, the findings mirror those in healthy participants.

##### Medium term studies

Four cross-over experiments lasted 2–30 d [50,76,91,108]. Neither high doses (45 mg/kg BW/d) nor low doses (15 mg/kg BW/d) of aspartame were found to affect blood glucose, insulin, or adverse events following supplementation for 20 d when compared with placebo or sucrose [108]. No effects of aspartame consumption for 2 wk were found in plasma glucose, insulin, insulin sensitivity, GLP-1, or leptin concentrations, when compared with sucralose [50]. No effects of aspartame with acesulfame K for 2 wk were found on glucose, insulin, or insulin sensitivity, when compared with mineral water [76]. Two weeks of aspartame supplementation also had no effects on fasting or postprandial blood glucose compared with no supplementation in individuals with DM [91].

##### Long term studies

Three cross-over experiments lasted for >30 d [56,64,96], with results that also mirror those above. Bonnet et al. [56] reported no effects of the consumption of aspartame with acesulfame K for 12 wk on glucose responses, insulin responses, insulin sensitivity, insulin secretion, or energy intake, compared with carbonated water. Colagiuri et al. [64] reported no effects of aspartame compared with sucrose for 6 wk on glucose, insulin, or measures of HbA1c in individuals with NIDDM, and Preechasuk et al. [96] reported no effects of aspartame compared with allulose for 12 wk on glucose, insulin, insulin sensitivity, insulin secretion, HbA1c, GLP-1, GIP, or adverse events in individuals with NIDDM.

#### Parallel-group studies

The 27 articles on parallel-group studies detailed 23 experiments, including 1 experiment which also included an exercise component [127].

##### Acute studies

Of the 22 nutritional experiments, 8 experiments were 1 d or less in duration, all conducted in healthy adults [122,126,136,137,[146], [147], [148]], providing between them 12 relevant studies. These 12 studies tested aspartame when administered alone (6 studies [126,136,146,148]), with other LCS (3 studies [122,137]), with nutritive components (2 studies [147]) or both (1 study [137]), and compared aspartame with glucose (8 studies [122,126,137,146,148]), sucrose (1 study [136]), and glucose and nutritive components (3 studies [137,147]). All studies assessed blood glucose levels (12 studies [122,126,136,137,[146], [147], [148]]), and 1 study assessed insulin [148] and glucagon [148]. Effects in glucose responses in these studies reflect those found in the acute cross-over studies. Aspartame was found to result in lower levels of blood glucose compared with glucose and sucrose consumption, whether provided alone, with other LCS, or with nutritive components. One study also reported on measures of appetite and adverse events in the form of hypoglycemia symptoms [136], to find no effects. The interventions, comparators, and outcomes in these studies are given in Table 2 [122,126,136,137,[146], [147], [148]].

**Table 2:** Parallel-groups nutritional studies of an acute duration, by intervention and comparator, to demonstrate outcomes assessed and effects of aspartame (↑ increase compared to comparator; ↓ decrease compared to comparator; ↔ no difference compared with comparator; NR – effects / results not reported).

Aspartame with …	Outcomes		
Comparator	Glucose	Insulin	Glucagon
**Alone**			
Glucose	4 studies ↓ [126,146] 1 study NR [148]	1 study NR [148]	1 study NR [148]
Sucrose	1 study ↓ [136]		
**Other NNS – Acesulfame K**			
Glucose	2 studies ↓ [122]		
**Other NNS – Saccharin**			
Glucose	1 study ↓ [137]		
**Nutritive**			
Glucose + Nutritive	2 studies ↓ [147]		
**Saccharin + Nutritive**			
Glucose + Nutritive	1 study ↓ [137]		

##### Medium term studies

Four experiments were 2–30 d in duration [130,139,145,149], one reported in multiple publications, each of which reports on select comparators and outcomes [129,130,142,143], with some inconsistencies in the effects reported depending on the studies included in each statistical analysis. Three experiments were conducted in healthy adults [130,139,145], where aspartame was provided alone and compared with sugars (6 studies [130]), provided with LCS and compared with other LCS (2 studies [139]) and water (1 study [139]), provided with glucose and compared with glucose alone (1 study [145]) and nothing (1 study [145]), and where aspartame was provided with high fructose corn syrup and compared with higher concentrations of high fructose corn syrup (2 studies [130]). Studies assessed glucose (13 studies [130,139,145]), HbA1c (5 studies [139,145]), insulin (13 studies [130,139,145]), insulin sensitivity (11 studies [130,139]), GLP-1 (5 studies [139,145]), and leptin (4 studies [130]). Effects in these studies mirror those in the short-term studies to some extent, where aspartame results in lower blood glucose and insulin levels and improved insulin sensitivity compared with sugars, with some variation between sugars, and some effects when aspartame was compared with other LCS, but effects are very inconsistent, and typically found in one measure only, where multiple measures were undertaken. The interventions, comparators, and outcomes in these studies are given in Table 3 [129,130,139,142,143,145]. Nine studies also reported on energy intake. The test situation differed for aspartame and comparator in 4 studies [130]; in the additional 5 studies, no effects were found [139,145]. Two studies also found no effects of aspartame on adverse events [145].

**Table 3:** Parallel-groups nutritional studies of a duration of 2 - 30 days, by intervention and comparator, to demonstrate outcomes assessed and effects of aspartame (↑ increase compared to comparator; ↓ decrease compared to comparator; ↔ no difference compared with comparator; NR – effects not reported).

Aspartame with …	Outcomes						
Comparator	Glucose	Insulin	HOMA-IR	Leptin	HbA1c	GLP-1	Matsuda Index
**Alone**							
Glucose	1 study ↓ [130] 1	1 study ↓ [130] 1	1 study ↔ [130]	1 study ↔ [130]			1 study ↔ [130] 2
Fructose	1 study ↑ [130], 1 study ↔ [130] 1	1 study ↑ [130], 1 study ↔ [130] 1	2 studies ↔ [130]	1 study ↑ [130]			1 study ↑ [130] 2
High Fructose Corn Syrup	2 studies ↓ [130] 1	1 study ↓ [130] 1 study ↔ [130] 1	2 studies ↔ [130]	1 study ↔ [130]			2 studies ↑ [130] 2
Sucrose	1 study ↔ [130]	1 study ↓ [130]	1 study ↔ [130]	1 study ↔ [130]			1 study ↑ [130] 2
**Other LCS – Acesulfame K**							
Saccharin	1 study ↓ [139] 3	1 study ↔ [139]	1 study ↔ [139]		1 study ↔ [139]	1 study ↔ [139]	1 study ↔ [139]
Sucralose	1 study ↔ [139]	1 study ↑ [139] 3	1 study ↔ [139]		1 study ↔ [139]	1 study ↔ [139]	1 study ↔ [139]
Water	1 study ↔ [139]	1 study ↔ [139]	1 study ↔ [139]		1 study ↔ [139]	1 study ↔ [139]	1 study ↔ [139]
**Sugars – Glucose**							
Glucose	1 study ↔ [145]	1 study ↔ [145]			1 study ↔ [145]	1 study ↔ [145]	
Nothing	1 study ↔ [145]	1 study ↔ [145]			1 study ↔ [145]	1 study ↔ [145]	
Saccharin + Glucose	1 study NR [145]	1 study NR [145]			1 study NR [145]	1 study NR [145]	
Sucralose + Glucose	1 study NR [145]	1 study NR [145]			1 study NR [145]	1 study NR [145]	
Stevia + Glucose	1 study NR [145]	1 study NR [145]			1 study NR [145]	1 study NR [145]	
**Sugars – High fructose corn syrup**							
High Fructose Corn Syrup	2 studies ↔ [130] 1	2 studies ↔ [130] 1	2 studies ↔ [130]				2 studies ↔ [130]

Outcomes –GLP-1: glucagon like peptide-1; HbA1c: Haemoglobin A1C (average blood glucose measures over the past 2-3 months); HOMA-IR: Homeostatic Model Assessment for Insulin Resistance; Note: some inconsistent effects are reported in articles 129,130,142,143, dependent on measure/s used and comparator/s used for analyses, and a tendency to report significant effects rather than all results; Analyses in article 145 are unclear, results are reported for analyses between each LCS vs glucose vehicle and nothing only.

1 similar, but some inconsistent effects found in Amplitudes of Glucose and Insulin responses;

2 similar, but some inconsistent effects reported in Predicted M Index, Stumvoll Index and Surrogate Hepatic IR Index; Some inconsistent effects reported in article 139, dependent on measure used;

3 effects found in some measures only.

One study was conducted in individuals with insulin-dependent diabetes mellitus (IDDM) [149], where aspartame was provided in snacks and compared with sucrose in snacks for 5 d. No differences were found between groups in blood glucose or fructosamine concentrations.

##### Long term studies

Ten experiments were >30 d in duration [123,124,128,[131], [132], [133], [134],^138^,^140^,^144^], 9 of which were conducted in healthy adults, one also involving children [134]. Experiments were noticeably larger with sample sizes ranging from 41 to 493 participants. In 3 experiments (7 studies) [124,132,134], aspartame was provided alone and compared with water/nothing (1 study [124]), 3 other LCS (3 studies [132]), sucrose or sucrose-sweetened drinks (2 studies [124,132]), and where aspartame was provided encapsulated, this was compared with encapsulated lactose (1 study [134]). In 4 experiments (6 studies), aspartame was provided with other LCS and compared with water/nothing (4 studies [123,128,133,140]) and sugar-sweetened drinks (2 studies [123,133]); in 1 experiment, aspartame was provided alongside other LCS in foods and beverages and compared with effects from the consumption of sucrose-sweetened foods and beverages (1 study [144]); and in 1 experiment (2 studies [131]), aspartame was provided with dextrose and compared with a dextrose vehicle. Studies assessed glucose (16 studies [123,124,128,[131], [132], [133], [134],^140^,^144^]), HbA1c (7 studies [128,131,132]), insulin (13 studies [123,124,128,131,132,134,144]), insulin sensitivity (5 studies [123,124,144]), leptin (4 studies [124,131]), 2 studies reported on GLP-1 [131] and GIP [131], and 1 study reported on glucagon [134]. None of these studies found differences between those consuming aspartame or a comparator in any biochemical measure. The interventions, comparators, and outcomes in these studies are given in Table 4 [123,124,128,[131], [132], [133], [134],^144^].

**Table 4:** Parallel-groups nutritional studies of a duration of > 30 days, by intervention and comparator, to demonstrate outcomes assessed and effects of aspartame (↑ increase compared to comparator; ↓ decrease compared to comparator; ↔ no difference compared with comparator; NR – effects not reported).

Aspartame with …	Outcomes								
Comparator	Glucose	Insulin	HOMA-IR / HOMA-%B / HOMA-%S	Leptin	HbA1c	Matsuda Index	GLP-1	GIP	Glucagon
**Alone**									
Water / Nothing	1 study ↔ [124]	1 study ↔ [124]	1 study ↔ [124]	1 study ↔ [124]		1 study ↔ [124]			
Saccharin	1 study ↔ [132]	1 study ↔ [132]			1 study ↔ [132]				
Sucralose	1 study ↔ [132]	1 study ↔ [132]			1 study ↔ [132]				
Rebaudioside A	1 study ↔ [132]	1 study ↔ [132]			1 study ↔ [132]				
Sucrose / Sucrose-sweetened drinks	2 studies ↔ [124,132]	2 studies ↔ [124,132]	1 study ↔ [124]	1 study ↔ [124]	1 study ↔ [132]	1 study ↔ [124]			
**Alone (encapsulated** [132]**)**									
Lactose	1 study ↔ [134]	1 study ↔ [134]							1 study ↔ [134]
**Other LCS – Non-specific**									
Water / Nothing	3 studies ↔ [123, 128, 140]	2 studies ↔ [123, 128]	1 study ↔ [123]		1 study ↔ [128]				
Sucrose-sweetened drinks	1 study ↔ [123]	1 study ↔ [123]	1 study ↔ [123]						
**Other LCS – Acesulfame K & Sucralose**									
Water / Nothing	1 study ↔ [133]								
Sucrose-sweetened drinks	1 study ↔ [133]								
**Other LCS + Nutritive – Acesulfame K, Cyclamate & Saccharin in foods & drinks**									
Sucrose-sweetened foods & drinks	1 study NR [144]	1 study NR [144]	1 study ↔ [144]						
**Carbohydrate / Dextrose**									
Dextrose vehicle	2 studies ↔ [131]	2 studies ↔ [131]		2 studies ↔ [131]	2 studies ↔ [131]		2 studies ↔ [131]	2 studies ↔ [131]	

Outcomes – GLP-1: glucagon like peptide-1; GIP: glucose dependent insulinotropic peptide; HbA1c: Haemoglobin A1C (average blood glucose measures over the past 2-3 months); HOMA-IR: Homeostatic Model Assessment for Insulin Resistance; HOMA-%B: Homeostatic Model Assessment for Beta-cell function; HOMA-%S: Homeostatic Model Assessment for Insulin Sensitivity.

Nine studies also provided data on discretionary energy intake [123,124,132,144], where either no differences were reported, or lower energy intake [123,144] and energy density [144] was reported in those consuming aspartame compared with sucrose, but no effects were found when compared with water [123]. Nine studies provided data on appetite [128,131,132,140,144] to report no differences between groups with the exceptions that those consuming aspartame self-reported lower hunger compared with those consuming water in the study by Peters et al. [140] and those consuming saccharin in the study by Higgins KA et al. [132]. Three studies reported no differences between groups in adverse events [123,134].

One experiment involved individuals with IDDM and NIDDM [138]. Here, encapsulated aspartame at high doses (2.7 g/d) was consumed for 18 wk, compared with encapsulated corn starch, to result in no changes in glucose metabolism, and comparable numbers of adverse events.

### Risk of Bias

Judgments of RoB for each included study, per outcome, are given in Supplemental Table 2. The majority of studies were considered to have "some concerns" over risk, predominantly as a result of concerns over randomization, concerns over effects of intervention assignment, and concerns over selected outcome reporting. Concerns over randomization largely arose in studies with a cross-over design, due to a lack of reporting of randomization processes in short term studies. Concerns over intervention assignment were predominantly a result of poor blinding or an inability to blind participants and researchers or outcome assessors to intervention assignment. Concerns over selected outcome reporting arose in studies with a cross-over design, as a result of the incomplete or unclear presentation of data and the incomplete or unclear reporting of statistical analyses, and in studies with a parallel-group design, as a result of the use of large trials with multiple outcomes, where time for analyses and space for reporting are limited, and/or outcomes are proposed for additional publications. Some concerns were also suggested where different comparisons and different outcomes have been reported in separate articles, or where analyses were unclearly reported. Studies without concerns over RoB were more often judged to have low RoB rather than high risk.

### Certainty of the evidence

Judgments of the certainty of the evidence for all primary outcomes are given in Table 5 for healthy populations, in Table 6 for populations with aspartame sensitivities, and in Table 7 for populations with compromised glucose metabolism. As already stated, wide heterogeneity in study methodology was found, resulting in the consideration of few studies per outcome depending on the provision of aspartame and comparator used. For all primary outcomes, the certainty of the evidence was considered to be “very low.” Certainty of the evidence was downgraded for limitations in study design and implementation, considering the concerns noted in the RoB assessments; inconsistency or heterogeneity in the evidence, considering the wide variation in study methodology, including the comparators used, and the significant differences found between subgroups in our meta-analyses; imprecision in the evidence available, considering the wide heterogeneity in study findings; and for some outcomes for possible risk of publication bias. For all outcomes, the majority of studies were deliberately designed to investigate our research questions, thus the certainty of the evidence was not downgraded for indirectness. For some outcomes, insufficient studies were available to estimate imprecision or other concerns. In these cases, the certainty of the evidence was again downgraded.

**Table 5:** Judgements of the certainty of the evidence for all primary outcomes, in healthy populations (lean, with overweight, with obesity), based on the GRADE criteria.

Certainty assessment							№ of participants		Effect		Certainty
№ of experiments	Study design	Risk of bias	Inconsistency	Indirectness	Imprecision	Other considerations	Aspartame	Any comparator	Relative (95% CI)	Absolute (95% CI)	
**Glucose responses, aspartame administered alone, < 1 day**											
23	cross-over studies	serious^a^	serious^b^	not serious	serious^c^	publication bias strongly suspected^d^	248	248	-	SMD **0.71 SD lower** (0.96 lower to 0.46 lower) Lower compared with sugars, carbohydartes or nutritive, no effects compared with vehicle or LCS.	⊕◯◯◯ Very low^a^^,^^b^^,^^c^^,^^d^
**Glucose responses, aspartame with nutritive element, < 1 day**											
14	cross-over studies	serious^a^	serious^b^	not serious	serious^c^	none	218	218	-	SMD **0.02 SD lower** (0.22 lower to 0.18 higher) No effects compared with sugars, nutritive, vehicle or LCS.	⊕◯◯◯ Very low^a^^,^^b^^,^^c^
**Glucose responses, all other administrations**											
17	cross-over studies	serious^a^	serious^b^	not serious	not estimable^e^	not estimable^e^	354	354	-	Lower compared with sugars, carbohydrates or nutritive, no effects compared with vehicle or LCS (acute). No effects in the medium- or long-term.	⊕◯◯◯ Very low^a^^,^^b^^,^^e^
20	parallel-groups studies	serious^a^	serious^b^	not serious	not estimable^e^	not estimable^e^	1131	1528	-	Lower compared with sugars or nutritive (acute). No or inconsistent effects in the medium-term. No effects in the long-term.	⊕◯◯◯ Very low^a^^,^^b^^,^^e^
**Insulin responses, aspartame administered alone, < 1 day**											
21	cross-over studies	serious^a^	serious^b^	not serious	serious^c^	publication bias strongly suspected^d^	214	214	-	SMD **1.12 SD lower** (1.62 lower to 0.62 lower) Lower compared with sugars, carbohydrates or nutritive, no effects compared with vehicle, higher compared with LCS.	⊕◯◯◯ Very low^a^^,^^b^^,^^c^^,^^d^
**Insulin responses, aspartame with nutritive element, < 1 day**											
13	cross-over studies	serious^a^	serious^b^	not serious	serious^c^	none	208	208	-	SMD **0.07 SD lower** (0.26 lower to 0.12 higher) Lower compared with sugars + nutritive, no effects compared with sugars, vehicle or LCS.	⊕◯◯◯ Very low^a^^,^^b^^,^^c^
**Insulin responses, all other administrations**											
17	cross-over studies	serious^a^	serious^b^	not serious	not estimable^e^	not estimable^e^	353	353	-	Lower compared with sugars, carbohydrates or nutritive, no effects compared with vehicle or LCS (acute). No effects in the medium- or long-term.	⊕◯◯◯ Very low^a^^,^^b^^,^^e^
11	parallel-groups studies	serious^a^	serious^b^	not serious	not estimable^e^	not estimable^e^	595	918	-	No or inconsistent effects in the medium-term. No effects in the long-term.	⊕◯◯◯ Very low^a^^,^^b^^,^^e^
**All other outcomes^1^, all administrations of aspartame**											
22	cross-over studies	serious^a^	serious^b^	not serious	not estimable^f^	not estimable^f^	466	466	-	No or inconsistent effects in acute. No effects in the medium- or long-term.	⊕◯◯◯ Very low^a^^,^^b^^,^^f^
11	parallel-groups studies	serious^a^	serious^b^	not serious	not estimable^f^	not estimable^f^	605	954	-	No or inconsistent effects in acute. No effects in the medium- or long-term.	⊕◯◯◯ Very low^a^^,^^b^^,^^f^

**CI:** confidence interval; **SMD:** standardised mean difference; ^1^ includes long-term measures of glucose levels (HbA1c, fructosamine) and measures of insulin sensitivity.

Explanations

a Some concerns in the majority of studies

b Wide variation in study methodology

c Wide heterogeneity between study findings

d Possible publication bias detected

e Not estimable given the low number of studies and wide variation in study methodology

f Not estimable given the wide variation in study methodology and variation in outcomes assessed

**Table 6:** Judgements of the certainty of the evidence for all primary outcomes, in aspartame-sensitive populations, based on the GRADE criteria.

Certainty assessment							№ of participants		Effect		Certainty
№ of experiments	Study design	Risk of bias	Inconsistency	Indirectness	Imprecision	Other considerations	Aspartame	Any comparator	Relative (95% CI)	Absolute (95% CI)	
**All outcomes, all administrations of aspartame**											
3	cross-over studies	serious^a^	serious^b^	not serious	not estimable^c^	not estimable^c^	100	100	-	No or inconsistent effects	⊕◯◯◯ Very low^a^^,^^b^^,^^c^

Explanations

a Some concerns in the majority of studies

b Wide variation in study methodology

c Not estimable given the low number of studies, wide variation in study methodology and variation in outcomes assessed

**Table 7:** Judgements of the certainty of the evidence for all primary outcomes, in populations with compromised glucose metabolism: mild, untreated, DM, NIDDM, IDDM, based on the GRADE criteria.

Certainty assessment							№ of participants		Effect		Certainty
№ of experiments	Study design	Risk of bias	Inconsistency	Indirectness	Imprecision	Other considerations	Aspartame	Any comparator	Relative (95% CI)	Absolute (95% CI)	
**All outcomes, all administrations of aspartame**											
9	cross-over studies	serious^a^	serious^b^	not serious	not estimable^c^	not estimable^c^	139	139	-	Lower compared with sugars or nutritive, no effects compared with vehicle or LCS (acute). No effects in the medium- or long-term.	⊕◯◯◯ Very low^a^^,^^b^^,^^c^
2	parallel-groups studies	serious^a^	serious^b^	not serious	not estimable^c^	not estimable^c^	37	41	-	No effects in the medium- or long-term.	⊕◯◯◯ Very low^a^^,^^b^^,^^c^

Explanations

a Some concerns in the majority of studies

b Wide variation in study methodology

c Not estimable given the low number of studies, wide variation in study methodology and variation in outcomes assessed

## Discussion

### Main findings

This work aimed to systematically identify and summarize all controlled intervention studies investigating the effects of aspartame consumption on glucose, insulin, and appetite-related hormone responses.

A considerable number of studies were identified, using wide variety in their methodology. Studies provided aspartame alone, with a range of other LCS, and with a range of nutritive sweeteners and other nutritive components, and compared the effects of aspartame consumption with placebo or vehicle, with a range of other LCS or a range of nutritive sweeteners or other nutritive components. Studies lasted from periods of <1 h to periods of ≤12 mo, and ranged in size from 4 to 493 participants, with necessary variety in aspartame provision and measurements taken. Almost all studies assessed effects on glucose and insulin responses, many of the longer (medium and long) term studies also included measures of insulin sensitivity (homeostatic model assessment for insulin resistance [HOMA-IR], Matsuda Index) and some studies included measures of long term glucose control (HbA1c), but few studies assessed other appetite-regulating hormones.

The variety in methodology in the studies available makes combination difficult. To allow meaningful results, meta-analyses were only conducted for acute cross-over studies that provided aspartame alone or with a nutritive element and investigated effects on blood glucose or insulin levels. These analyses demonstrate no effects of aspartame when compared with vehicle for either glucose or insulin, and these limited responses then also result in lower blood glucose and insulin levels when compared with nutritive substances. Slight differences in glucose and insulin responses in some subgroups most likely reflect the different studies in each subgroup and differences in the methods used for both aspartame administration and outcome assessment. An absence of effect was also found when aspartame was compared with other LCS; some small effects were found in insulin responses, but very few studies could be included in these analyses.

Caution must be exercised in relation to our meta-analyses considering the limited studies included, the assumptions and estimations required, and the high heterogeneity that was found. Some suggestion of publication bias was found in our funnel plots, and other sources of heterogeneity could not be investigated due to the limited studies available, but the short term effects reported were largely also found in the studies that did not contribute to the meta-analyses, and similar effects were also found in the acute studies using parallel-group designs. With these considerations, the certainty of the evidence for glucose and insulin outcomes over short periods was judged to be “very low.”

In the medium and long term studies, few effects of aspartame consumption were found. In the medium term, some effects mirrored those found over the short term to some extent, but significant differences were less consistent. There is a suggestion again that the effects of aspartame may differ from those of other LCS, but again very few studies were considered. Given the different chemical structures and metabolic actions of different LCS [19,22,23], further investigation in this area may be of value.

In the long term studies, no effects of the repeated consumption of aspartame were found on any of the measures assessed. This absence of effects most likely reflects the different measures used in these studies, and, importantly, the long term nature of these parameters. Measures of glycosylated hemoglobin (HbA1C) reflect glucose metabolism over months, will be unaffected by immediate food intake as assessed in the short term, and are more closely related to chronic health conditions [150,151]. These findings suggest no contraindications for long term glucose metabolism from aspartame consumption. The certainty of the evidence for all outcomes over the medium and long term, however, was judged to be “very low.” Natural variation between individuals, diets, and dietary patterns will lessen the chances of observing effects, as will a backdrop of a usual diet composed of a range of sugars and LCS onto which aspartame is added, and any changes in dietary behavior that may occur in response to an intervention. In many of the long term studies, furthermore, the intervention was less controlled [123,128,133,140], and an assessment, specifically of aspartame, may have been compromised. In studies where the consumption specifically of aspartame was more closely controlled [131,132,134], however, again, no meaningful effects were found.

Few studies measured appetite-regulating hormones other than insulin. Leptin, GLP-1, and GIP were measured in some studies, and again few effects were found in these outcomes, particularly over the long term. Some work, however, does suggest differential effects of different LCS on a number of appetite-regulating hormones, specifically GLP-1 and GIP [23]; further work in this area would be of value, particularly over the long term. Only two long term [124,131] and three medium term experiments [130,139,145] that assessed appetite-regulating hormones other than insulin were found. These studies further all provided different exposures to aspartame and used different comparators. The certainty of the evidence for all appetite-regulating hormone responses other than insulin was judged to be “very low.”

Of interest, a lack of effects from aspartame was also found, not only in healthy individuals but also in those with self-reported aspartame sensitivity and in those with compromised glucose metabolism in the form of DM. Our findings may suggest again few reasons for concern over aspartame consumption, but few studies with these populations were available, and the physiology underlying DM is complicated by diverse forms of the condition, comorbidities, and other confounders [[150], [151], [152]]. The certainty of the evidence for all outcomes in specific populations was judged to be “very low.”

Some studies also assessed energy intake and appetite alongside effects on blood chemistry. Effects in these outcomes typically mirrored those found in the blood in the short term — no effects when compared with vehicle or other LCS, and reduced energy intake and appetite following aspartame when compared with sugars or other nutritive components, with few differences between interventions in the long term. These findings have also been demonstrated in studies that do not measure biochemistry [13,14,153,154]. Where adverse events were assessed, no effects of aspartame consumption were found.

### Comparisons with other reviews

Other reviews on this topic also report no effects of aspartame when compared with water/vehicle, although when compared with sugars, findings are mixed. Ahmad et al. [34] reviewed 18 articles that studied the provision of aspartame on glucose metabolism and appetite-regulating hormones, compared with vehicle and sugars, to report limited effects of aspartame, although the high heterogeneity between studies, particularly in study methodology, was also noted. This review further includes 2 articles we were unable to access [155,156]. According to the reports by Ahmad et al. [34], both these acute studies found no effects of aspartame on glucose [155,156] or insulin [155] compared with both sucrose [155,156] and other LCS [155], in healthy adults [155] and in adults with NIDDM [156]. Mehat et al. [35] in their review also reported limited effects of aspartame when combined with acesulfame K on blood glucose and other appetite-regulating hormones, when compared with water and sugars, although few studies are included, and again high heterogeneity was noted. Our review includes considerably more studies than were included in either of these previous reviews.

Reviews on LCS more generally also suggest limited glucose and insulin responses from a range of LCS [[31], [32], [33]] and lower blood glucose and insulin levels when compared with sugars. Of these, where LCS have been separated, Greyling et al. [31] reported no effects of aspartame alone or with other nutritive elements on postprandial glucose or insulin responses when compared with vehicle. Zhang et al. [33] also reported no effects of aspartame, when provided alone and in combination with other LCS when compared with vehicle, and reduced effects when compared with nutritive components. The review by Zhang et al. [33] provides similar findings for a range of other appetite-regulating hormones in response to other LCS.

### Limitations of the review

Our review is limited by the small number of studies with comparable methods, making combination difficult, the low number of medium and long term studies, and the low number of studies measuring appetite-regulating hormones other than insulin. Test of aspartame is difficult further in the medium term and long term given the prevalence of aspartame in the food supply, responses in these studies that are potentially affected by a range of other elements in food products, e.g., beverage flavorings and preservatives, and hormone assessment that is expensive and possibly compromised by practical issues. Heterogeneity between all studies was very high, and although insufficient comparable studies were available to investigate sources, we do find some evidence of publication bias in our funnel plots.

Our review processes may also have been compromised. Our search processes were extensive, but some studies may still have been missed if specific terms were not mentioned by authors. We included a range of terms related to LCS to capture articles on aspartame and attempted to contact authors, but further studies may have been suitable unbeknownst to authors, e.g., through provision of “a diet drink,” particularly as a placebo comparator for a sugar-rich drink, and not all authors replied to our requests. Appetite-regulating hormones similarly may have been incorporated under much broader terms, e.g., “metabolic biomarkers,” so these studies may have been missed. We also did not search for unpublished work, except via trial registries and conference abstracts. Our searches of trial registrations and conference abstracts, however, did result in the addition of a small number of studies that would not otherwise have been included. Many studies also failed to report composite measures of hormone responses requiring calculations and estimations for group SDs, and none of the cross-over studies reported the correlation between data points within subjects for the different intervention arms. Full data would have enabled increased accuracy in our analyses [43] and may also have allowed the combination of cross-over and parallel-group studies in the same analyses, enhancing the number of studies included, and so the power of these [157]. Many estimations were required for our meta-analyses, and caution must be exercised here.

### Implications for practice

Caution must be exercised considering the very low certainty of the evidence available. Notwithstanding also the aforementioned limitations, the findings of this review suggest few impacts of aspartame consumption on appetite-regulating hormones, with potential benefits for glucose metabolism, energy intake, and appetite when compared with the consumption of sugars, and no detrimental contraindications following consumption in the long term. Increasing research demonstrates benefits for LCS when compared with the consumption of sugars [13,14,153,154]. Concerns have been expressed over their use in the long term following associations with a number of health conditions [30,31], but direct causal impacts are difficult to ascertain in the research available [3,19,158]. Recent reviews confirm the safety of aspartame for human consumption [[25], [26], [27]], but further work on health implications over the long term would be of value.

### Implications for research

Additional studies, particularly over the medium and long term, would increase the evidence base and the certainty of the evidence. Indeed, considering the consistency required for “moderate” or “high” certainty evidence, many additional studies may be required before implications for practice can be made responsibly. Further studies over all study durations would aid in understanding the heterogeneity found in our results, and more nuanced reviews will be of interest once a fuller evidence base is achieved, e.g., based on time frame. Investigations by BW, habitual LCS use, or other personal characteristics may also be of interest. Further work on the effects of aspartame on a wider range of appetite-regulating hormones would be of value, with a focus on proposed mechanisms [22,23,116,131,132]. Some value may also be gained from consideration of hormones that influence appetite only indirectly, e.g., epinephrine. Also of potential interest are the possible differences in effects from different LCS. Studies where LCS are directly compared suggest some slight differences between LCS [29,132,139], likely as a result of their different chemical structures and metabolic fates [22,23]. It will be important, however, to ensure studies remain representative of everyday use, e.g., in dose, consumption patterns, in considering combinations of LCS, and in reference to the overall diet.

### Conclusions

In conclusion, we found a considerable number of studies of relevance to our research questions, but these studies varied greatly in the methodology used, and the certainty of the evidence for all outcomes in all populations was considered to be “very low.” The majority of studies investigated blood glucose and insulin levels over the short term, and meta-analyses of these studies reveal no effects of aspartame when compared with vehicle or other LCS, and found lower blood glucose and insulin levels following aspartame compared with sugars or other nutritive components. Medium and long term studies demonstrate few effects of aspartame consumption regardless of comparator. Few medium and long term studies, however, were found, and few studies assessed appetite-regulating hormones other than insulin. Further investigation of aspartame in comparison with other LCS would also be of value.

## Acknowledgments

We are grateful to all article authors who responded to our emails, allowing the inclusion of greater evidence or greater confidence in the evidence included.

## Author contributions

The authors’ responsibility were as follows – KMA: formulated the research questions, developed the protocol and registered the review on PROSPERO; LRB, FE, JW, ADB: undertook all searches, screening and data extraction; KMA: undertook all primary analyses, and wrote the first draft of the paper; and all authors have read, reviewed, edited and agree with the final version of the article.

## Data availability

Data described and not available in the manuscript, code book, and analytic code will be made available upon reasonable request of the corresponding author.

## Funding

The work presented in this article was funded by Ajinomoto Health and Nutrition North America, Inc., United States. The funder played no role in undertaking the work. LRB, FE, JW, ADB also had no contact with the funder.

## Conflict of interest

For work in the area of sweet taste and low-calorie sweeteners, in the past 3 y, KMA has previously received research funding from the International Sweeteners Association, BE. She has current funding from a consortium of the American Beverage Association, Arla Foods, Cargill R&D Centre Europe BVBA, DSM-Firmenich SA, International Sweeteners Association, SinoSweet Co., Ltd, Cosun Nutrition Center and Unilever Foods Innovation Centre Wageningen, and from The Coca Cola Company, United States. She has received speaker’s expenses from the International Sweeteners Association, BE; the CBC group, Israel, and EatWell Global. All other authors have no conflicts to declare.
