# Supplementary material for: The Effects of Aspartame on Glucose, Insulin, and Appetite-Regulating Hormone Responses in Humans: Systematic Review and Meta-Analyses
Source: Adv Nutr. 2025 May 15;16(7):100449. doi: 10.1016/j.advnut.2025.100449 (PMC12205327; doi:10.1016/j.advnut.2025.100449)
Supplement: Multimedia component 2 [file mmc2.docx]

**THE EFFECTS OF ASPARTAME ON GLUCOSE, INSULIN AND APPETITE-REGULATING HORMONE RESPONSES IN HUMANS: SYSTEMATIC REVIEW AND META-ANALYSES**

Boxall et al.

**SUPPLEMENTARY MATERIALS**

**METHODS**

**Search terms used for searches for all electronic databases**

Terms relating to aspartame: (aspartam* OR E-951 OR equal OR Nutrasweet OR “Sugar Twin” OR aspartame-sweetened OR low-calorie (sweet* OR food* OR beverage* OR drink* OR “soft drink” OR soda* OR solution) OR “low calorie” (sweet* OR food* OR beverage* OR drink* OR “soft drink” OR soda* OR solution) OR low-caloric (sweet* OR food* OR beverage* OR drink* OR “soft drink” OR soda* OR solution) OR “low caloric” (sweet* OR food* OR beverage* OR drink* OR “soft drink” OR soda* OR solution) OR zero-calorie OR “zero calorie” OR low-energy (sweet* OR food* OR beverage* OR drink* OR “soft drink” OR soda* OR solution) OR “low energy” (sweet* OR food* OR beverage* OR drink* OR “soft drink” OR soda* OR solution) OR non-nutritive (sweet* OR food* OR beverage* OR drink* OR “soft drink” OR soda* OR solution) OR nonnutritive (sweet* OR food* OR beverage* OR drink* OR “soft drink” OR soda* OR solution) OR “non nutritive” (sweet* OR food* OR beverage* OR drink* OR “soft drink” OR soda* OR solution) OR nonsugar (sweet* OR food* OR beverage* OR drink* OR “soft drink” OR soda* OR solution) OR non-sugar (sweet* OR food* OR beverage* OR drink* OR “soft drink” OR soda* OR solution) OR “non sugar” (sweet* OR food* OR beverage* OR drink* OR “soft drink” OR soda* OR solution) OR non-caloric (sweet* OR food* OR beverage* OR drink* OR “soft drink” OR soda* OR solution) OR noncaloric (sweet* OR food* OR beverage* OR drink* OR “soft drink” OR soda* OR solution) OR artificially-sweetened OR “artificially sweetened” OR low-calorie-sweetened OR zero-calorie-sweetened OR diet (beverage* OR drink* OR “soft drink” OR soda*) OR NNS OR NSS.

Terms searched for in ‘title’ OR ‘abstract’ fields.

AND

Terms relating to glucose, insulin, or appetite-regulating hormone responses: (Glucose OR insulin* OR glycemic OR glycaemic OR glucagon OR OGTT OR HOMA OR HOMA-IR OR HbA1C OR Haemaglobin A1C OR Hemaglobin A1C OR “glycated haemaglobin” OR “glycated hemaglobin” OR fructosamine OR C-peptides OR “C peptides” OR cholecystokinin OR CCK OR ghrelin OR “pancreatic tyrosine tyrosine” OR PYY OR peptide-YY OR “peptide YY” OR “glucagon-like peptide-1” OR “glucagon like peptide-1” OR “glucagon like peptide 1” OR “glucagon-like peptide 1” OR GLP-1 OR “GLP 1” OR “glucose-dependent insulinotropic peptide” OR “glucose-dependent insulinotropic-peptide” OR “glucose-dependent-insulinotropic- peptide” OR “glucose dependent insulinotropic peptide” OR GIP OR oxyntomodulin OR obestatin OR nesfatin OR leptin OR adiponectin OR incretin* OR “gut hormone*” OR “gastrointestinal hormone*” OR “gastro-intestinal hormone*” OR “appetite-regulating” OR “appetite-regulation” OR “gut peptide*” OR “gastric inhibitory peptide” OR “gastric inhibitory polypeptide” OR gastrin).

Terms searched for in ‘title’ OR ‘abstract’ fields.

**RESULTS**

**Excel File: Full study methodology details are given in the Excel File**

File includes all details per references and for all references included in the review, regardless of whether these are subsequently included in analyses. Details that were not reported in articles are signified with NR – not reported. Where details are noted as NR – with a detail added, this detail was not explicitly reported, but has concluded based on the evidence that is reported, e.g. if total number of participants is reported, and number of males, number of females, while not reported, has been calculated.

**RESULTS**

**Supplementary Table 1:** Cross-over nutritional studies of an acute (<1 day) duration, by intervention and comparator, to demonstrate outcomes assessed.

| **Aspartame with …** | | **Outcomes** | | | | | | | | | |
| --- | --- | --- | --- | --- | --- | --- | --- | --- | --- | --- | --- |
|  | **Comparator** | Glucose | Insulin | Glucagon | Ghrelin | GLP-1 | GIP | Additional | Energy Intake | Appetite | Adverse events |
| **Alone** | | | | |  |  |  |  |  |  |  |
|  | Vehicle (Water / Gum / Flavoured drink) | 58; 58; 59; 73; 87; 104; 120 | 58; 58; 59; 73; 87; 104; 120 | 59; 73 |  |  |  |  |  |  | 73 |
|  | Vehicle + Glucose | 54; 54; 70; 70; 75; 89; 91; 97; 104; 121 | 54; 54; 75; 89; 91; 97; 104; 121 | 91; 97; 121 |  |  |  |  | 97 |  |  |
|  | Vehicle + Sucrose | 59; 83; 117 | 59; 83; 117 | 59 | 83 | 83 | 83 |  | 83; 117 | 83; 117 |  |
|  | Vehicle + Fructose | 97 | 97 | 97 |  |  |  |  | 97 |  |  |
|  | Vehicle + Glucose & Fructose | 86 |  |  |  |  |  |  |  |  |  |
|  | Vehicle + Maltodextrin / dextrose | 101; 101; 101; 104 | 101; 101; 101; 104 |  |  |  |  |  |  |  |  |
|  | Vehicle + Nutritive | 62; 74; 74; 87; 90; 90; 120 | 62; 74; 74; 87; 90; 90; 120 |  |  |  |  |  |  | 62 |  |
|  | Vehicle + Saccharin | 73 | 73 | 73 |  |  |  |  |  |  | 73 |
|  | Vehicle + Stevia | 117 | 117 |  |  |  |  |  | 117 | 117 |  |
|  | Vehicle + Monk Fruit | 117 | 117 |  |  |  |  |  | 117 | 117 |  |
|  | Vehicle + d-allulose | 77 | 77 |  |  |  |  |  |  |  |  |
|  | Vehicle + Erythritol |  |  |  | 107 |  |  |  |  | 107 |  |
| **Alone not tasted (encapsulated [58;69], after Gymnema Sylvestre [73] or intragastric [108])** | | | | |  |  |  |  |  |  |  |
|  | Aspartic acid | 60 | 60 |  |  |  |  |  |  |  |  |
|  | Phenylalanine | 60; 60 | 60; 60 |  |  |  |  |  |  |  |  |
|  | Aspartic Acid & Phenylalanine | 71 | 71 |  |  | 71 | 71 | 71^#^ |  | 71 |  |
|  | Cornflour | 71 | 71 |  |  | 71 | 71 | 71^#^ |  | 71 |  |
|  | Glucose | 75; 110 | 75; 110 | 110 | 110 | 110 |  | 110* |  | 110 | 110 |
|  | Fructose | 110 | 110 | 110 | 110 | 110 |  | 110* |  | 110 | 110 |
|  | Water | 110 | 110 | 110 | 110 | 110 |  | 110* |  | 110 | 110 |
|  | Acesulfame K | 110 | 110 | 110 | 110 | 110 |  | 110* |  | 110 | 110 |
|  | Sucralose | 110 | 110 | 110 | 110 | 110 |  | 110* |  | 110 | 110 |

| **Alone not tasted (encapsulated [69,79]) & nutritive** | | | | |  |  |  |  |  |  |  |
| --- | --- | --- | --- | --- | --- | --- | --- | --- | --- | --- | --- |
|  | Aspartic Acid & Phenylalanine + nutritive | 71 | 71 |  |  | 71 | 71 | 71^#^ |  | 71 |  |
|  | Cornflour + nutritive | 71 | 71 |  |  | 71 | 71 | 71^#^ |  | 71 |  |
|  | Placebo + nutritive | 81 |  |  |  |  |  |  |  | 81 | 81 |
| **Alone tasted, not ingested (sip & spit [113])** | | | | |  |  |  |  |  |  |  |
|  | Vehicle | 115; 115 | 115; 115 |  |  |  |  |  |  |  |  |
|  | Vehicle + Sucrose | 115; 115 | 115; 115 |  |  |  |  |  |  |  |  |
|  | Vehicle + Saccharin | 115; 115 | 115; 115 |  |  |  |  |  |  |  |  |
|  | Nutritive | 115; 115 | 115; 115 |  |  |  |  |  |  |  |  |
| **Alone tasted, not ingested (sham-fed [112]) & nutritive** | | | | |  |  |  |  |  |  |  |
|  | Nutritive vehicle + salt | 114; 114 | 114; 114 |  |  |  |  | 114**; 114** |  |  |  |
| **Other LCS – Saccharin** | | | | |  |  |  |  |  |  |  |
|  | Vehicle + CHO | 119 |  |  |  |  |  |  |  |  |  |
| **Other LCS – Acesulfame K + Sodium cyclamate** | | | | |  |  |  |  |  |  |  |
|  | Vehicle |  | 67 |  |  |  |  |  |  |  |  |
|  | Vehicle + Sucrose | 105 | 67; 67; 105 |  | 105 | 105 |  |  |  | 105 |  |
|  | Vehicle + High fructose corn syrup | 105 | 105 |  | 105 | 105 |  |  |  | 105 |  |
| **Other LCS – Acesulfame K + glucose** | | | | |  |  |  |  |  |  |  |
|  | Sucrose | 106 |  |  |  |  |  |  |  |  |  |
| **Other LCS – Acesulfame K + Sucralose & Nutritive** | | | | |  |  |  |  |  |  |  |
|  | Vehicle + (additional) Sucralose | 112 | 112 |  |  | 112 | 112 |  |  | 112 |  |
| **Glucose** | | | | |  |  |  |  |  |  |  |
|  | Vehicle | 57; 116 | 116 |  |  | 116 |  |  |  | 57 |  |
|  | Vehicle + acesulfame K | 57 |  |  |  |  |  |  |  | 57 |  |
|  | Vehicle + saccharin | 57 |  |  |  |  |  |  |  | 57 |  |
|  | Vehicle + sucralose | 116 | 116 |  |  | 116 |  |  |  |  |  |
| **Sucrose** | | | | |  |  |  |  |  |  |  |
|  | Vehicle | 59 | 59 | 59 |  |  |  |  |  |  |  |
| **Carbohydrate / Dextrose** | | | | |  |  |  |  |  |  |  |
|  | Vehicle | 49; 58; 120 | 49; 58; 120 | 49 |  |  |  |  |  |  |  |
|  | Vehicle + Sucrose | 102 | 102 |  |  |  |  |  |  |  |  |
|  | Sucrose | 49 | 49 | 49 |  |  |  |  |  |  |  |
| **Carbohydrate / Dextrose & Sucrose** | | | | |  |  |  |  |  |  |  |
|  | Vehicle | 102 | 102 |  |  |  |  |  |  |  |  |
| **Nutritive** | | | | |  |  |  |  |  |  |  |
|  | Nutritive vehicle | 95; 98; 103 | 95; 98; 103 |  |  | 98 | 98 | 98*** |  | 95; 98 | 98 |
|  | Nutritive vehicle + Stevia | 53 | 53 |  |  |  |  |  | 53 | 53 | 53 |
|  | Nutritive vehicle + Sucrose | 53; 85; 94 | 53; 85; 94 |  |  |  |  |  | 53 | 53; 85; 94 | 53 |
|  | Nutritive vehicle + Glucose | 103 | 103 |  |  |  |  |  |  |  |  |
|  | Nutritive vehicle + Fructose | 103 | 103 |  |  |  |  |  |  |  |  |
|  | Nutritive vehicle + d-allulose | 77 | 77 |  |  |  |  |  |  |  |  |
|  | Sucrose | 95 | 95 |  |  |  |  |  |  | 95 |  |

Numbers signify references to articles. Where numbers are repeated separated with a semi-colon, each number denotes a different experiment within the same article, a different dose of aspartame, a different vehicle, or a different dose of the same comparator. Alone comparisons that test the effects of aspartame separate from those of other substances, vehicles or context-specific variations are included. Where studies include multiple comparisons that may be of interest, all comparisons have been included as separate studies. References 65, 84, 93 are not included because the nutritive elements varied between conditions. References 109, 110, 118 are not included because other aspects of the intervention were also missing in comparator.

Outcomes – GLP-1: glucagon like peptide-1; GIP: glucose dependent insulinotropic peptide;

Other Outcomes – ^#^ CCK: Cholescystokinin; * PYY: Polypeptide Tyrosine Tyrosine; ** PP – Pancreatic Polypeptide; *** HOMA-IR: Homeostatic Model Assessment for Insulin Resistance.

**RESULTS**

**Meta-Analysis 1: Aspartame alone, Glucose Responses**

**Analyses using fixed effects models**

Meta-analysis 1 included 34 cross-over studies, all providing aspartame alone, divided into 5 subgroups dependent on comparator (vehicle, sweet-tasting sugars, non-sweet-tasting or non-specific CHO, nutritive components, other LCS); suitable data were not available for blood glucose for inclusion from four studies [83,107,120]. Using fixed effects models, effects of aspartame were found when compared with vehicle (SMD = -0.44, 95% CI: -0.91, 0.03, I^2^ = 37%, 6 studies), no effects were found when compared with other LCS (SMD = 0.10, 95% CI: -0.28, 0.48, I^2^ = 0%, 4 studies), and significantly lower levels of blood glucose were found following aspartame when compared with sweet-tasting sugars (SMD = -0.81, 95% CI: -1.06, -0.57, I^2^ = 55%, 14 studies), non-sweet-tasting/non-specific CHO (SMD = -1.33, 95% CI: -2.08, -0.58, I^2^ = 0%, 4 studies) and other nutritive components (SMD = -1.04, 95% CI: -1.55, -0.54, I^2^ = 0%, 6 studies). Statistically significant differences were found between subgroups (χ^2^ = 22.98, p < .01). The overall effect (SMD = -0.63, 95% CI: -0.80, -0.46, I^2^ = 50%, 34 studies) reflects the subgroups and studies involved.

**Supplementary** **Figure 1:** Funnel Plot for meta-analysis 1 (34 studies)


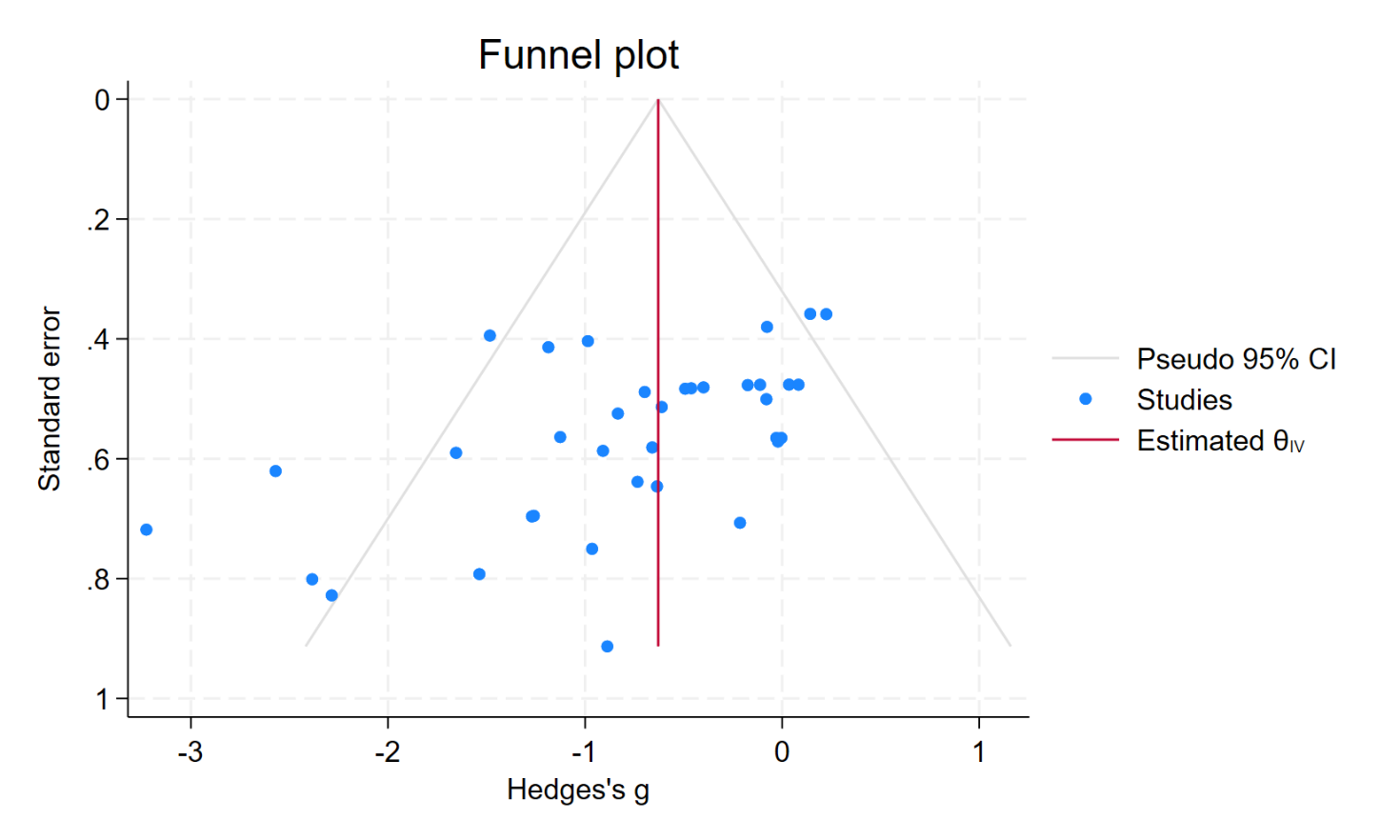


**RESULTS**

**Meta-Analysis 2: Aspartame with a nutritive component, Glucose Responses**

**Supplementary Figure 2:** Forest Plot for meta-analysis 2 (random effects models) investigating the effects of aspartame administered with a nutritive element on glucose responses in healthy individuals (cross-over studies). Individual studies are represented by the blue boxes, combined effects are represented by the diamonds. Studies on the 0 line demonstrate no differences between aspartame and comparator, studies to the right of the 0 line demonstrate greater responses to aspartame, studies to the left of the 0 line demonstrate reduced responses to aspartame / increased responses to comparator.


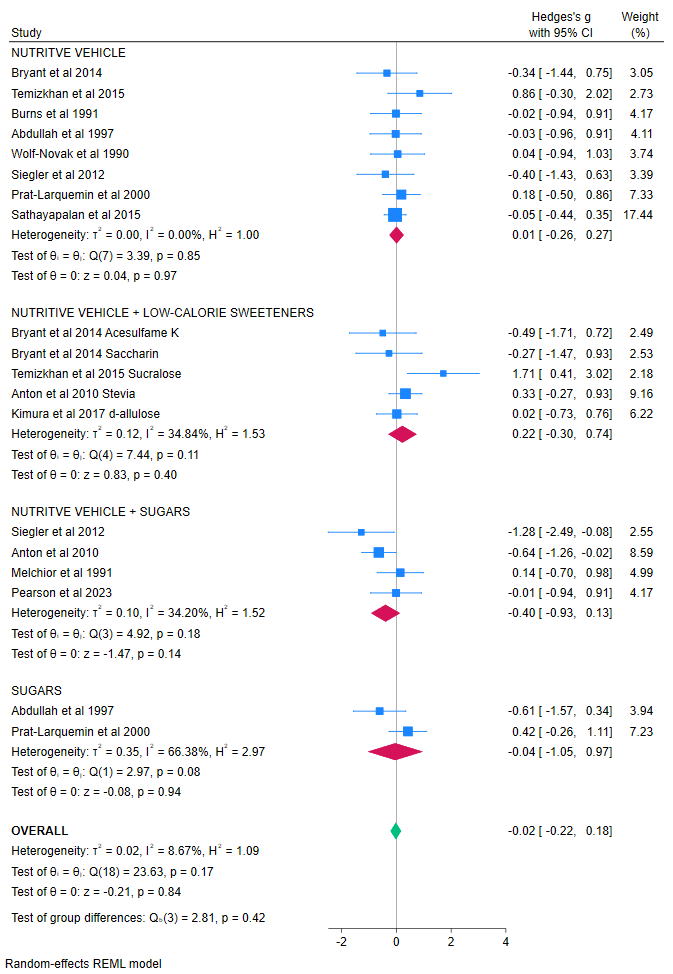


**Analyses using fixed effects models**

Meta-analysis 2 included 19 cross-over studies, all providing aspartame with a nutritive element, divided into 3 subgroups dependent on comparator (nutritive vehicle, nutritive vehicle with LCS, nutritive vehicle with nutritive sugars or other elements, sugars); suitable data were not available for blood glucose for inclusion from four studies [58,103]. Using fixed effects models, no effects of aspartame were found when compared with nutritive vehicle (SMD = 0.01, 95% CI: -0.26, 0.27, I^2^ = 0%, 8 studies), nutritive vehicle and LCS (SMD = 0.22, 95% CI: -0.17, 0.61, I^2^ = 46%, 5 studies), nutritive vehicle and sugars (SMD = -0.40, 95% CI: -0.82, 0.01, I^2^ = 39%, 4 studies), or sugars (SMD = 0.07, 95% CI: -0.49, 0.63, I^2^ = 66%, 2 studies). No statistically significant differences were found between subgroups (χ^2^ = 4.90, p = 0.18), and the overall effect (SMD = -0.02, 95% CI: -0.20, 0.16, I^2^ = 24%, 19 studies) demonstrates no effects.

**Supplementary Figure 3:** Funnel Plot for meta-analysis 2 (19 studies)


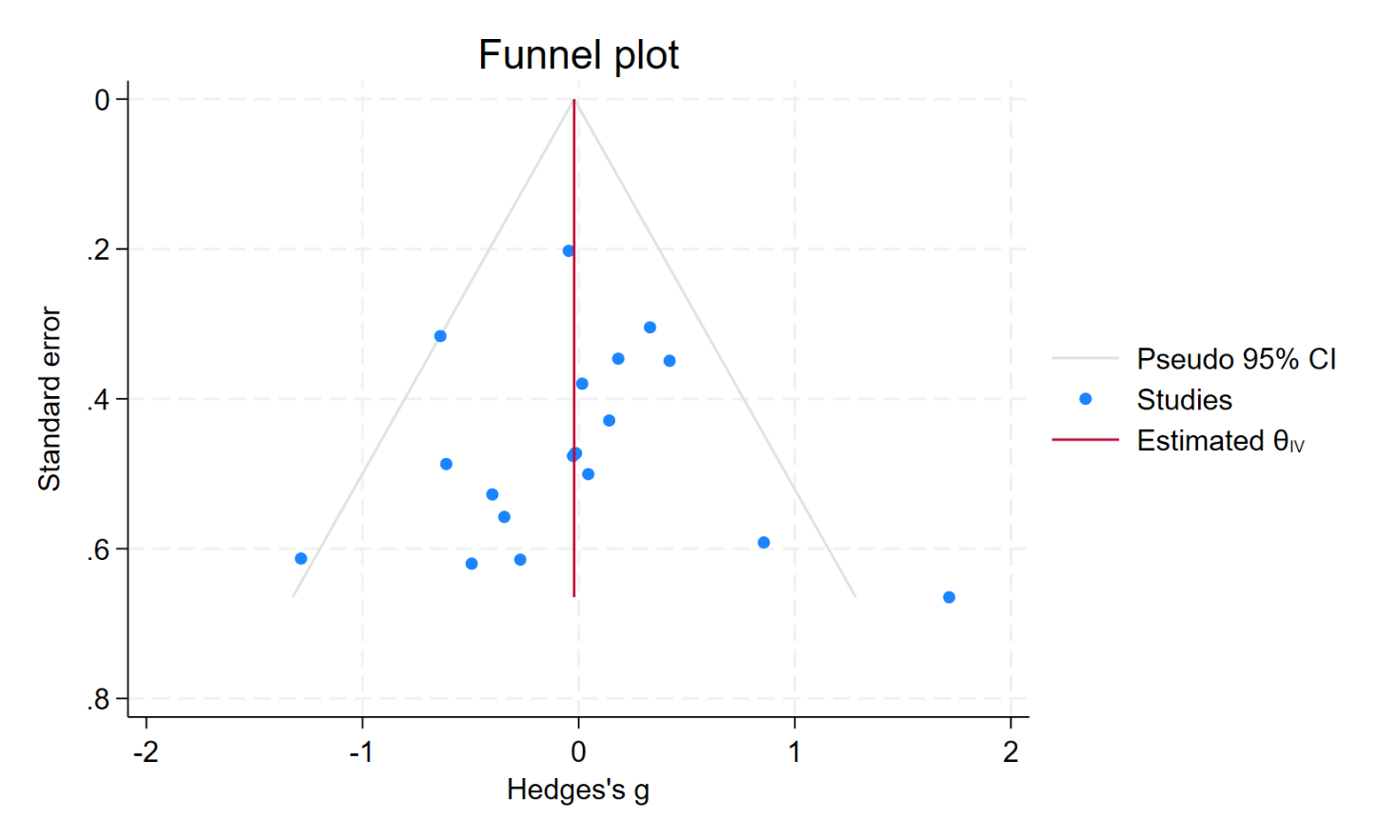


**RESULTS**

**Meta-Analysis 3: Aspartame alone, Insulin Responses**

**Analyses using fixed effects models**

For insulin responses, meta-analysis 3 included 31 cross-over studies, all providing aspartame alone, divided into 5 subgroups dependent on comparator (vehicle, sweet-tasting sugars, non-sweet-tasting or non-specific CHO, nutritive components, other LCS); suitable data were not available for blood insulin for inclusion from seven studies [70,83,86,107,120]. Using fixed effects models, no effects of aspartame were found when compared with vehicle (SMD = 0.04, 95% CI: -0.42, 0.49, I^2^ = 0%, 6 studies), significantly lower levels of blood insulin were found following aspartame when compared with sweet-tasting sugars (SMD = -1.41, 95% CI: -1.73, -1.10, I^2^ = 83%, 11 studies (2 population sub-groups), non-sweet-tasting/non-specific CHO (SMD = -2.00, 95% CI: -2.82, -1.17, I^2^ = 12%, 4 studies) and other nutritive components (SMD = -1.65, 95% CI: -2.22, -1.09, I^2^ = 46%, 6 studies), and significantly higher levels of blood insulin were found following aspartame when compared with other LCS (SMD = 0.71, 95% CI: 0.31, 1.10, I^2^ = 66%, 4 studies). Statistically significant differences were found between subgroups (χ^2^ = 99.54, p < .01). The overall effect (SMD = -0.68, 95% CI: -0.87, -0.48, I^2^ = 83%, 31 studies) represents the majority of studies included.

**Supplementary Figure 4:** Funnel Plot for meta-analysis 1 (random effects models)


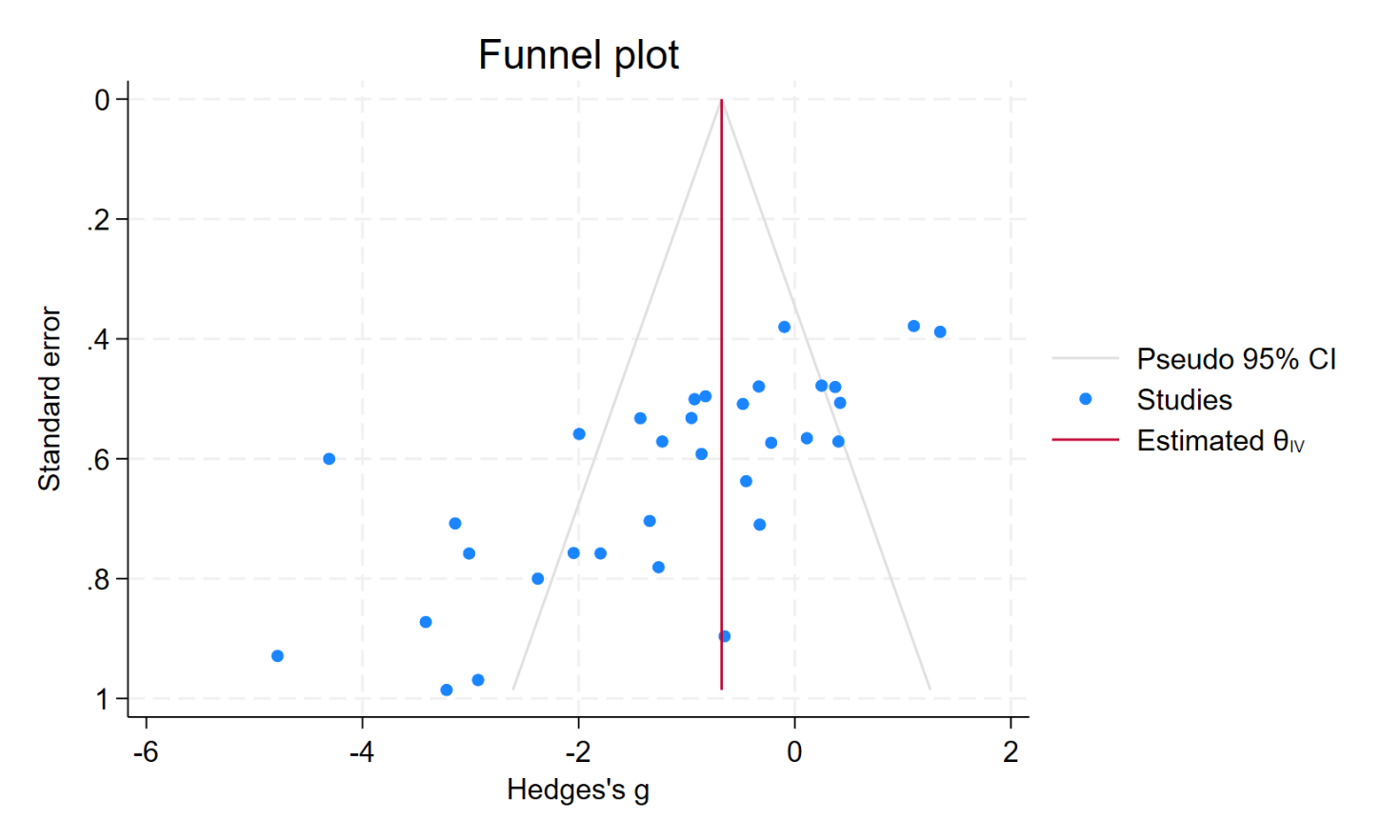


**RESULTS**

**Meta-Analysis 4: Aspartame with a nutritive component, Insulin Responses**

**Supplementary Figure 5:** Forest Plot for meta-analysis 4 (random effects models) investigating the effects of aspartame administered with a nutritive element on insulin responses in healthy individuals (cross-over studies). Individual studies are represented by the blue boxes, combined effects are represented by the diamonds. Studies on the 0 line demonstrate no differences between aspartame and comparator, studies to the right of the 0 line demonstrate greater responses to aspartame, studies to the left of the 0 line demonstrate reduced responses to aspartame / increased responses to comparator.

**
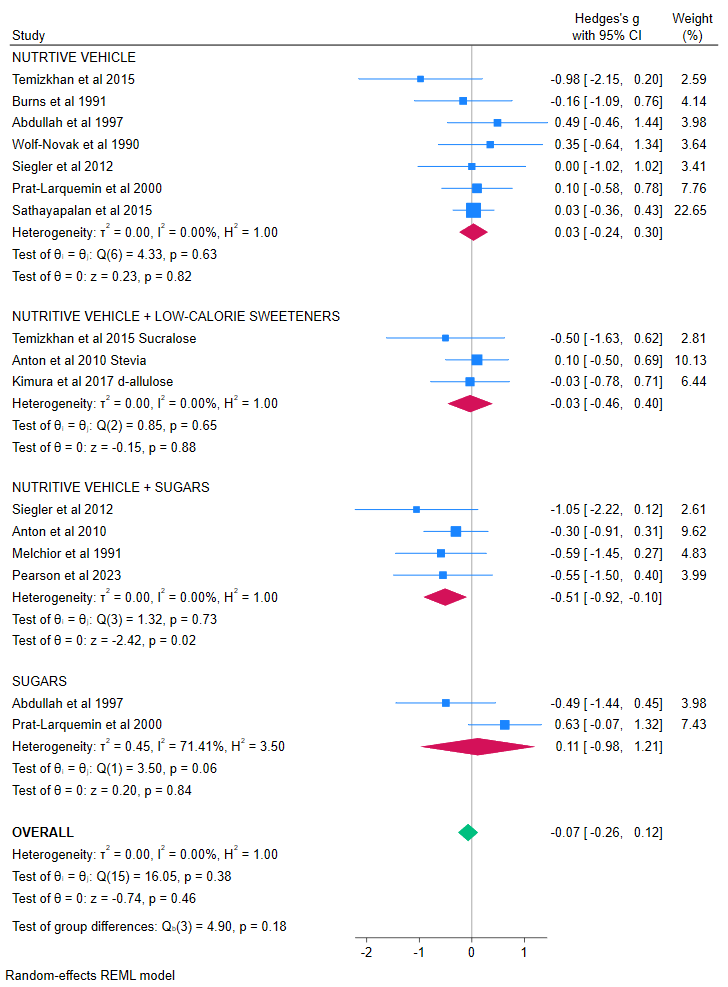
**

**Analyses using fixed effects models**

Meta-analysis 4 included 16 cross-over studies, all providing aspartame with a nutritive element, that were divided into four subgroups dependent on comparator (nutritive vehicle, nutritive vehicle with LCS, nutritive vehicle with nutritive sugars or other elements, sugars); suitable data were not available for blood insulin for inclusion from seven studies [57,58,103]. Using fixed effects models, no effects of aspartame were found when compared with nutritive vehicle (SMD = 0.03, 95% CI: -0.24, 0.30, I^2^ = 0%, 7 studies), nutritive vehicle and LCS (SMD = -0.03, 95% CI: -0.46, 0.40, I^2^ = 0%, 3 studies), or sugars (SMD = 0.24, 95% CI: -0.32, 0.80, I^2^ = 71%, 2 studies), but a significantly lower blood insulin following aspartame was found when compared with nutritive vehicle and sugars (SMD = -0.51, 95% CI: -0.92, -0.10, I^2^ = 0%, 4 studies). Differences between subgroups were not statistically significant (χ^2^ = 6.05, p = 0.11). The overall effect (SMD = -0.07, 95% CI: -0.26, 0.12, I^2^ = 7%, 16 studies) represents the majority of the studies in the analyses.

**Supplementary Figure 6:** Funnel Plot for meta-analysis 1 (16 studies)


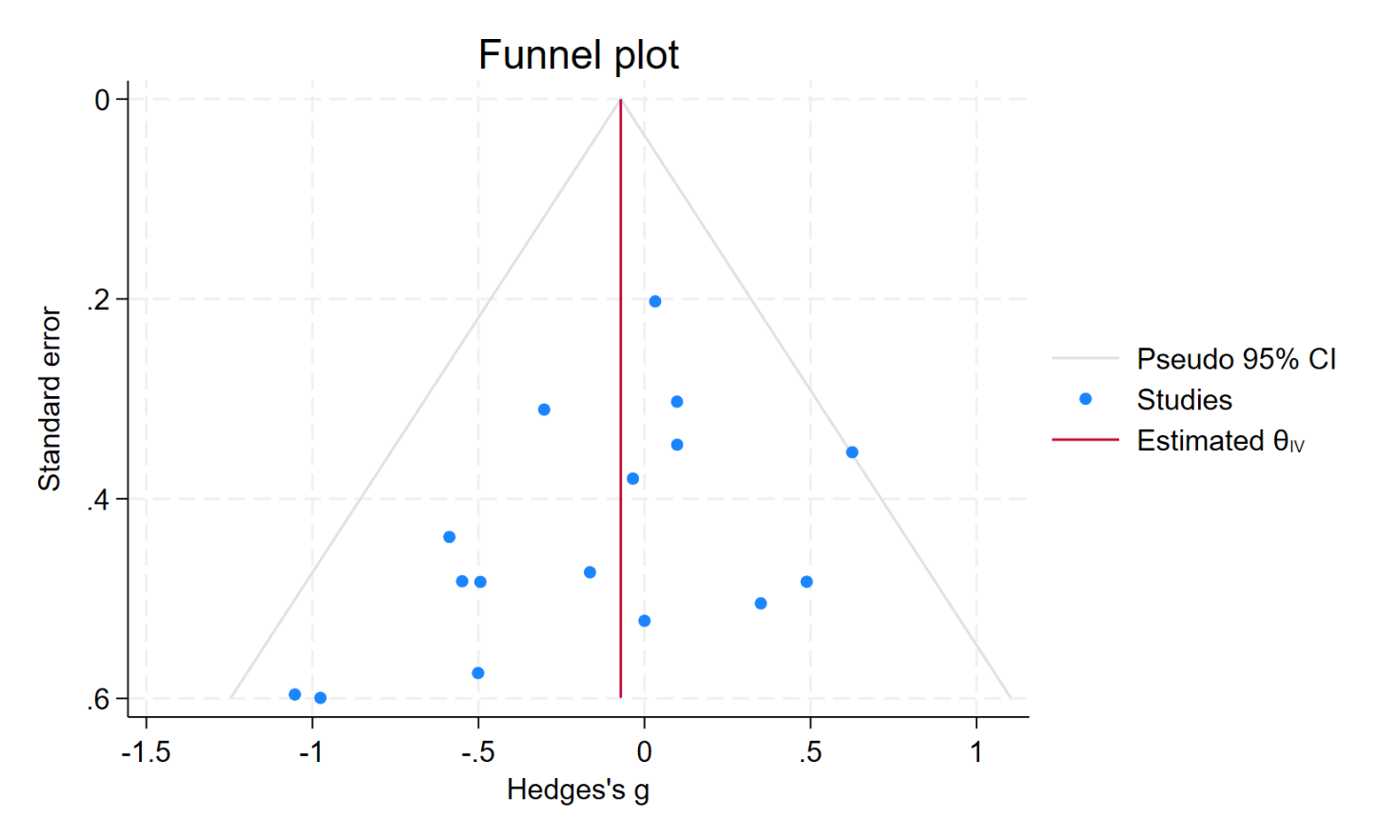


**Supplementary Table 2:** Risk of bias for all included studies. Judgements are given, per outcome, for all domains assessed and an overall assessment given, where “low” denotes low risk of bias, “high” denotes high risk of bias, and “concerns” denotes some concerns.

| Author | Outcomes | 1. Randomisation | 2. Deviations from intended interventions (effect of assignment) | 2. Deviations from intended interventions (effects of adherence) | 3. Missing Outcome Data | 4. Measurement of the outcome | 5. Selection of reported result/s | Overall Bias | |
| --- | --- | --- | --- | --- | --- | --- | --- | --- | --- |
| Studies with a Crossover Design | | | | | | | | | |
| Abdallah et al 1997 [49] | Glucose | Concerns | Low | Low | High | Low | Concerns | High | |
|  | Insulin, Glucagon | Concerns | Low | Low | Low | Low | Concerns | Concerns | |
| Ahmad et al 2020 [50] | Glucose, Insulin, GLP-1, Leptin, HOMA-IR, HOMA-%B, HOMA-%S | Low | Low | Low | Low | Low | Low | Low | |
|  | Fructosamine | Low | Low | Low | High | Low | Low | High | |
| Akalp et al 2023 [51] | Glucose | Low | Concerns | Low | Low | Low | Concerns | Concerns | |
| Ali et al 2016 [52] | Glucose, insulin | Concerns | Concerns | Concerns | Low | Low | Concerns | Concerns | |
| Anton et al 2010 [53] | Glucose, insulin, energy intake, adverse events | Concerns | Concerns | Low | Low | Low | Concerns | Concerns | |
|  | Appetite | Concerns | Concerns | Low | Low | Concerns | Concerns | Concerns | |
| Berlin et al 2005 [54] | Glucose, insulin | Low | Low | Low | Low | Low | Concerns | Concerns | |
| Bird et al 2013 [55] | Glucose | Concerns | Concerns | Low | Low | Low | Concerns | Concerns | |
| Bonnet et al 2018 [56] | Glucose, Insulin, Matsuda Index, HOMA-IR, Insulinogenic Index, Disposition Index, Stumvoll Indices, Energy Intake | Concerns | Low | Low | Low | Low | Low | Concerns | |
| Bryant et al 2014 [57] | Glucose | Concerns | Concerns | Low | Low | Low | Concerns | Concerns | |
|  | Appetite – fullness | Concerns | Concerns | Low | Low | Concerns | Concerns | Concerns | |
| Bruce et al 1987 [58] Experiment 2 | Glucose, insulin | Concerns | Concerns | Low | Low | Low | Concerns | Concerns | |
| Bruce et al 1987 [58] Experiment 3 | Glucose, insulin | Concerns | Concerns | Low | Low | Low | Concerns | Concerns | |
| Burns et al 1991 [59] | Glucose, insulin, glucagon | Concerns | Concerns | Low | Low | Low | Concerns | Concerns | |
| Carlson and Shah 1988 [60] | Glucose, insulin | Concerns | Low | Low | Low | Low | Concerns | Concerns | |
| Chong et al 2014 [61] | Glucose | Concerns | Low | Low | Low | Low | Concerns | Concerns | |
| Chryssanthopoulos et al 2008 [62] | Glucose, insulin, appetite - fullness | Concerns | Concerns | Low | Low | Low | Concerns | Concerns | |
| Coggan and Coyle 1989 [63] | Glucose, insulin | Concerns | Concerns | Low | Low | Low | Concerns | Concerns | |
| Colagiuri et al 1989 [64] | Glucose, insulin, HbA1c | Concerns | Low | Low | Low | Low | Concerns | Concerns | |
| Cuomo et al 2011 [65] | Glucose, CCK, Ghrelin, Energy intake | Low | Low | Low | Low | Low | Concerns | Concerns | |
|  | Appetite, Adverse events | Low | Low | Low | Low | Concerns | Concerns | Concerns | |
| Fahey et al 1991 [66] | Glucose | Concerns | Concerns | Low | Low | Low | Concerns | Concerns | |
| Finassi et al 2023 [67] | Insulin | Concerns | Concerns | Low | Low | Low | Low | Concerns | |
| Fukuda et al 2010 [68] | Glucose, insulin, adverse events | Concerns | Concerns | Low | Low | Low | Concerns | Concerns | |
| Gam et al 2014 [69] | Glucose | Concerns | Concerns | Low | Low | Low | Concerns | Concerns | |
| Green et al 2001 [70] | Glucose | Concerns | High | High | Low | Low | Concerns | High | |
| Hall et al 2003 [71] | Glucose, insulin, GIP, GLP-1 | Concerns | Concerns | Low | Low | Low | Concerns | Concerns | |
|  | Appetite | Concerns | Concerns | Low | Low | Concerns | Concerns | Concerns | |
| Hargreaves & Briggs 1988 [72] | Glucose, insulin | Concerns | Concerns | Low | Low | Low | Concerns | Concerns | |
| Horwitz et al 1988 [73] | Glucose, insulin, glucagon | Low | Concerns | Low | Low | Low | Concerns | Concerns | |
| Karamanolis et al 2011 [74] | Glucose, insulin | Concerns | Concerns | Low | Low | Low | Concerns | Concerns | |
| Kashima et al 2019 [75] | Glucose, insulin | Concerns | Concerns | Low | Low | Low | Concerns | Concerns | |
| Kim et al 2020 [76] | Glucose, insulin, HOMA-IR, OGTT Stumvoll Index, OGTT Matsuda Index | Low | Concerns | Low | High | Low | Low | High | |
| Kimura et al 2017 [77] | Glucose, insulin | Concerns | Low | Low | Low | Low | Concerns | Concerns | |
| Kingwell et al 1989 [78] | Glucose | Concerns | Low | Low | Low | Low | Concerns | Concerns | |
| Koch et al 2001 [79] | Glucose | Low | Low | Low | Low | Concerns | Concerns | Concerns | |
| Kumar et al 2019 [80] | Glucose | Low | Concerns | Low | Low | Low | Concerns | Concerns | |
| Lapierre et al 1990 [81] | Glucose, appetite, adverse events | Concerns | Concerns | Low | Low | Low | Concerns | Concerns | |
| Lehmann et al 2021 [82] | Glucose | Concerns | Concerns | High | Low | Low | Low | High | |
| Maersk, Belza, Holst, et al 2012 [83] | Glucose, insulin, ghrelin, GLP-1, GIP, Energy intake | Low | Concerns | Low | Low | Low | Concerns | Concerns | |
|  | Appetite | Low | Concerns | Low | Low | Concerns | Concerns | Concerns | |
| Melanson et al 1999 [84] | Glucose, energy intake, appetite (all day) | Concerns | Concerns | Concerns | Low | Low | Concerns | Concerns | |
| Melchior et al 1991 [85] | Glucose, insulin, appetite | Concerns | Low | Low | Low | Low | Concerns | Concerns | |
| Millard-Stafford et al 1992 [86] | Glucose | Concerns | Low | Low | Low | Low | Concerns | Concerns | |
| Moller 1991 [87] | Glucose, insulin | Concerns | Concerns | Low | Low | Low | Concerns | Concerns | |
| Nassis et al 1998 [88] | Glucose | Concerns | Low | Low | Low | Low | Concerns | Concerns | |
| Nguyen et al 1998 [89] | Glucose, insulin | Concerns | Low | Low | Low | Low | Concerns | Concerns | |
| Noriega et al 1997 [90] Experiment 2 | Glucose, insulin | Concerns | Low | Low | Low | Low | Concerns | Concerns | |
| Okuno et al 1986 [91] Single administration | Glucose, insulin, glucagon | Concerns | Low | Low | Low | Low | Concerns | Concerns | |
| Okuno et al 1986 [91] Continuous administration | Glucose | Concerns | Low | Low | Low | Low | Concerns | Concerns | |
| Osterberg et al 1985 [92] | Glucose, insulin | Concerns | Concerns | Low | Low | Low | Concerns | Concerns | |
| Panahi et al 2013 [93] | Glucose, Energy intake | Concerns | Concerns | Low | Low | Low | Concerns | Concerns | |
|  | Appetite | Concerns | Concerns | Low | Low | Concerns | Concerns | Concerns | |
| Pearson et al 2023 [94] | Glucose, Appetite | Concerns | Concerns | Low | Low | Low | Concerns | Concerns | |
|  | Insulin | Concerns | Concerns | Low | Low | Low | Concerns | Concerns | |
| Prat-Larquemin et al 2000 [95] | Glucose, insulin, appetite | Concerns | Concerns | Low | Low | Low | Concerns | Concerns | |
| Preechasuk et al 2023 [96] | Glucose, insulin, HOMA-IR, HOMA-%B, HbA1c, GLP-1, GIP, Matsuda Index, Insulinogenic Index, Adverse events | Low | Low | Low | Low | Low | Concerns | Concerns | |
| Rodin 1990 [97] | Lean - Glucose, insulin, glucagon, energy intake | Concerns | Low | Low | Low | Low | Concerns | Concerns | |
|  | Overweight - Glucose, insulin, glucagon, energy intake | Concerns | Low | Low | Low | Low | Concerns | Concerns | |
| Sathyapalan et al 2015 [98] | Glucose, insulin, HOMA-IR, GLP-1, GIP, appetite, adverse events | Low | Low | Low | Low | Low | Low | Low | |
| Schiffman et al 1987 [99] | Glucose, insulin, glucagon, adverse events | Concerns | Concerns | Low | Low | Concerns | Concerns | Concerns | |
| Shigeta et al 1985 [100] Experiment 2a | Glucose, insulin | Concerns | Concerns | Low | Concerns | Concerns | Concerns | Concerns | |
| Short et al 1997 [101] | Glucose, insulin | Concerns | Low | Low | Low | Low | Concerns | Concerns | |
| Siegler et al 2012 [102] | Glucose, insulin | Concerns | Concerns | Low | Low | Low | Concerns | Concerns | |
| Singleton et al 1999 [103] | Glucose, insulin | Concerns | Concerns | Low | Low | Low | Concerns | Concerns | |
| Smeets et al 2005 [104] | Glucose, insulin | Low | Low | Low | Low | Low | Concerns | Concerns | |
| Soenen and Westerterp-Plantenga 2007 [105] Experiment 1 | Glucose, Insulin, GLP-1, Ghrelin | Concerns | Concerns | Low | Low | Low | Concerns | Concerns | |
|  | Appetite | Concerns | Concerns | Low | Low | Concerns | Concerns | Concerns | |
| Solomi et al 2019 [106] | Glucose | Concerns | Concerns | Low | Low | Low | Concerns | Concerns | |
| Sorrentino et al 2020 [107] | Ghrelin, Appetite | Low | Low | Low | Low | Low | Concerns | Concerns | |
| Spiers et al 1998 [108] | Glucose, insulin, adverse events | Low | Low | Low | Low | Low | Concerns | Concerns | |
| Stannard et al 2000 [109] | Glucose | Concerns | Concerns | Low | Low | Low | Concerns | Concerns | |
| Steinert et al 2011 [110] Full Study | Glucose, insulin, GLP-1, Ghrelin, PYY, Glucagon, Appetite, Adverse events | Concerns | Low | Low | Low | Low | Concerns | Concerns | |
| Sturm et al 2004 [111] | Glucose, insulin, CCK | Low | Concerns | Low | Low | Low | Concerns | Concerns | |
|  | Energy intake, appetite | Low | Concerns | Low | Low | Concerns | Concerns | Concerns | |
| Sylvetsky et al 2016 [112] Study Arm 2 | Glucose, insulin, GLP-1, GIP | Low | Concerns | Low | Low | Low | Concerns | Concerns | |
|  | Appetite | Low | Concerns | Low | Low | Concerns | Concerns | Concerns | |
| Tamis-Jortberg et al 1996 [113] | Glucose, insulin | Concerns | Concerns | Low | Low | Concerns | Concerns | Concerns | |
| Teff 2010 [114] Experiment 3 | Glucose, insulin, Pancreatic polypeptide | Concerns | Concerns | Concerns | Low | Low | Concerns | Concerns | |
| Teff et al 1995 [115] Experiment 1 | Glucose, insulin | Low | Concerns | Low | Low | Low | Concerns | Concerns | |
| Teff et al 1995 [115] Experiment 2 | Glucose, insulin | Low | Concerns | Low | Low | Low | Concerns | Concerns | |
| Temizkan et al 2015 [116] | Glucose, insulin, GLP-1 | Concerns | Concerns | Low | Low | Low | Concerns | Concerns | |
| Tey et al 2017 [117] | Glucose, insulin, energy intake, appetite | Low | Low | Low | Low | Low | Low | Concerns | |
| Warwick et al 1993 [118] | Glucose, energy intake, appetite | Concerns | Concerns | Low | Low | Low | Concerns | Concerns | |
| Wax et al 2013 [119] | Glucose | Low | Low | Low | Low | Low | Concerns | Concerns | |
| Wolf-Novak et al 1990 [120] | Glucose, insulin | Concerns | Low | Low | Low | Low | Concerns | Concerns | |
| Wouassi et al 1997 [121] | Glucose, insulin, glucagon | Concerns | Low | Low | Low | Low | Concerns | Concerns | |
| Studies with a Parallel-groups Design | | | | | | | | | |
| Benton & Owens 1993 [122] Experiment 1 | Glucose | Concerns | High | Low | Low | Concerns | Concerns | High |  |
| Benton & Owens 1993 [122] Experiment 2 | Glucose | Concerns | High | Low | High | Concerns | Concerns | High |  |
| Ebbeling et al 2020 [123] | Glucose, insulin, HOMA-%B, HOMA-%S, energy intake, adverse events | Concerns | High | Concerns | Low | Concerns | Low | Concerns |  |
| Engel et al 2018 [124, 125] | Glucose, insulin, HOMA-IR, Matsuda Index, Leptin, energy intake | Concerns | High | Concerns | Concerns | Low | Concerns | Concerns |  |
| Maersk et al [135] | Glucose, insulin, HOMA-IR, Matsuda Index, leptin, energy intake | Concerns | Concerns | Concerns | Concerns | Low | Concerns | Concerns |  |
| Finley et al 2019 [126] | Glucose | Concerns | High | Low | High | Low | Concerns | High |  |
| Gozal et al 1985 [127] | Glucose, insulin, glucagon | Concerns | Concerns | Low | Low | Low | Concerns | Concerns |  |
| Harrold et al 2024 [128] | Glucose, insulin, HbA1c | Low | High | Low | Low | Low | Low | High |  |
|  | Appetite - hunger | Low | High | Low | Low | Concerns | Low | High |  |
| Hieronimus et al 2024 [130] | Glucose, Insulin, HOMA-IR, Matsuda Index, Predicted M Index, Stumvoll Index, Surrogate hepatic IR Index, Leptin, energy intake | Concerns | Concerns | Low | Low | Low | Concerns | Concerns |  |
| Hieronimus et al 2020 [129] | Glucose, Insulin, HOMA-IR, Matsuda Index, Predicted M Index, Stumvoll Index, Surrogate hepatic IR Index, Leptin, energy intake | Concerns | Concerns | Low | Low | Low | Concerns | Concerns |  |
| Sigala, et al 2022 [142] | Glucose, Insulin, HOMA-IR, Matsuda Index, Predicted M Index, Stumvoll Index, Surrogate hepatic IR Index, Leptin, energy intake | Concerns | Concerns | Low | Concerns | Low | Concerns | Concerns |  |
| Sigala, et al 2020 [143] | Glucose, Insulin, HOMA-IR, Matsuda Index, Predicted M Index, Stumvoll Index, Surrogate hepatic IR Index, Leptin, energy intake | Concerns | Concerns | Low | Concerns | Low | Concerns | Concerns |  |
| Higgins et al 2018 [131] | Glucose, insulin, HbA1c, GLP-1, GIP, leptin, appetite | Low | Concerns | Low | Low | Low | Low | Concerns |  |
| Higgins and Mattes 2019 [132] | Glucose, insulin, HbA1c, energy intake, appetite - sucrose comparator | Low | Low | Low | Concerns | Low | Concerns | Concerns |  |
|  | Glucose, insulin, HbA1c, energy intake, appetite – other comparators | Low | Low | Low | Low | Low | Concerns | Concerns |  |
| Kendig et al 2023 [133] | Glucose | Concerns | Concerns | Low | Low | Low | Low | Concerns |  |
| Knopp et al 1976 [134] | Glucose, insulin, glucagon, adverse events | Concerns | Concerns | Low | Low | Low | Concerns | Concerns |  |
| Markus and Rogers 2020 [136] Experiment 1 | Glucose - sucrose comparator | Concerns | Low | Low | Concerns | Low | Concerns | Concerns |  |
|  | Glucose - milk comparator | Concerns | Concerns | Low | Concerns | Low | Concerns | Concerns |  |
|  | Appetite, adverse events - sucrose comparator | Concerns | Low | Low | Concerns | Concerns | Concerns | Concerns |  |
|  | Appetite, adverse events - milk comparator | Concerns | Concerns | Low | Concerns | Concerns | Concerns | Concerns |  |
| Martin and Benton 1999 [137] | Glucose | Concerns | Concerns | Low | Low | Low | Concerns | Concerns |  |
| Nehrling et al 1985 [138] | Glucose, glycohaemoglobin (HbA1c), adverse events | Concerns | Low | Low | Low | Low | Concerns | Concerns |  |
| Orku et al 2023 [139] | Glucose, insulin, HOMA-IR, HbA1c, GLP-1, Matsuda Index, energy intake | Low | Concerns | Low | Low | Low | Low | Concerns |  |
| Peters et al 2016 [140] | Glucose | Low | Low | Low | Low | Low | Concerns | Concerns |  |
|  | Appetite – hunger | Low | Low | Low | Low | Concerns | Concerns | Concerns |  |
| Sorensen et al 2005 [144] | Glucose, insulin, HOMA-IR, energy intake | Concerns | Concerns | Low | Low | Low | Concerns | Concerns |  |
|  | Appetite | Concerns | Concerns | Low | Low | Concerns | Concerns | Concerns |  |
| Raben et al 2002 [141] | Glucose, insulin, HOMA-IR, energy intake | Concerns | Concerns | Low | Low | Low | Concerns | Concerns |  |
|  | Appetite | Concerns | Concerns | Low | Low | Concerns | Concerns | Concerns |  |
| Suez et al 2022 [145] | Glucose, insulin, GLP-1, HbA1c, Energy intake, Adverse events | Concerns | Concerns | Low | Concerns | Low | Concerns | Concerns |  |
| Sunram-Lea et al 2001 [146] | Glucose | Low | Low | Low | Low | Low | Concerns | Concerns |  |
| Sunram-Lea et al 2004 [147] | Glucose | Low | Concerns | Low | Low | Low | Concerns | Concerns |  |
| Virkkunen et al 1994 [148] | Glucose, insulin, glucagon | Concerns | Concerns | Low | Low | Low | Concerns | Concerns |  |
| Wise et al 1989 [149] | Glucose, fructosamine | Concerns | Concerns | Concerns | Low | Low | Concerns | Concerns |  |

Outcomes – CCK: cholesystokinin; GLP-1: glucagon like peptide-1; GIP: glucose dependent insulinotropic peptide; HbA1c: Haemoglobin A1C (average blood glucose measures over the past 2-3 months); HOMA-%B: Homeostatic Model Assessment for Beta-cell function; HOMA-IR: Homeostatic Model Assessment for Insulin Resistance; HOMA-%S: Homeostatic Model Assessment for Insulin Sensitivity; OGTT: Oral Glucose Tolerance Test; Predicted M Index: PYY: Polypeptide Tyrosine Tyrosine.
